# A Behavioral and Biological Analysis of Aesthetics: Implications for Research and Applications

**DOI:** 10.1007/s40732-017-0228-1

**Published:** 2017-06-19

**Authors:** Francis Mechner

**Affiliations:** 1 Columbia University, 116th St & Broadway, New York, NY 10027 USA; 2 The Mechner Foundation, 200 Central Park South #18e, New York, NY 10019 USA

**Keywords:** Experimental aesthetics, Synergetics, Concept analysis, Priming, Aesthetics, Art, Beauty, Culture, Emotion, Humor, Literature, Mirroring, Music, Neuroscience, Parsimony, Poetry, Reinforcement, Symmetry, Empirical semantics and fuzzy concepts

## Abstract

Seeking to identify the common and distinguishing attributes of effects one might call “aesthetic,” I examined hundreds of examples in music, visual arts, poetry, literature, humor, performance arts, architecture, science, mathematics, games, and other disciplines. I observed that all involve quasi-emotional reactions to stimuli that are composites of multiple elements that ordinarily do not occur together and whose interaction, when appropriately potentiated, is transformative—different in kind from the effects of the separate constituent elements. Such effects, termed *synergetic,* can evoke surprise-tinged emotional responses. Aesthetic reactions, unlike many other kinds of emotional reactions, are never evoked by biologically urgent action-demanding events, such as threats or opportunities. The examined effects were created by various concept manipulation devices: class expansion, identification of new relations, repetition, symmetry, parsimony, and emotional displays for the audience to mirror (I identified a total of 16 such devices). The effects would occur only for individuals with the necessary priming, in circumstances that include effective potentiating factors. Synergetic stimuli that evoke aesthetic responses tend to be reinforcing, via mechanisms related to their biological utility during our evolution. I offer a theory as to how aesthetics may have evolved from its primordial pre-aesthetic roots, with examples of how consideration of those roots often explains aesthetic and related effects. The article suggests that aesthetic phenomena are a special case of a more pervasive aspect of behavior and proposes research approaches involving laboratory models and fMRI technology.

TABLE OF CONTENTS

- Part 1 The Quest For a Theory
- 1.1 A Provocative Phenomenon
- 1.2 What Is Special About the Stimulus?
- 1.3 The Aesthetic Response
- 1.4 Operant Components
- 1.5 Applications Beyond Aesthetics
- 1.6 Synergetic Interaction: Nature’s Master Key
- 1.7 Synergetic Interactions in Behavior
- 1.8 The Synergetic Brew
- 1.9 Synergetic Effects in the Arts
- 1.10 Devices that Create Effective Synergetic Brews
- 1.11 Potentiation and Priming of Aesthetic Responses
- 1.12 Common Features of Aesthetic Phenomena
- 1.13 A Diagrammatic Overview
- 1.14 Synergistic Versus Synergetic Interactions
- 1.15 How the Present Approach Differs
- 1.16 The Domain of Emotional Reactions
- 1.17 Research Models of Aesthetic Phenomena
- 1.18 Research Implications for Sub-Aesthetic Effects
- Part 2 The Central Concept of Concept
- 2.1 How the Term is Used
- 2.2 Nonverbal Noncognitive Concepts
- 2.3 The Concept Repertoire
- 2.4 Concepts and Their Relations
- 2.5 Why Concepts are Central to the Theory
- Part 3 Evolutionary Roots of Aesthetic Phenomena
- 3.1 Emotion-Evoking Effects of Synergetic Brews
- 3.2 A Theory of How Aesthetic Responses Evolved
- 3.3 Evolution of the Beauty Concept
- 3.4 The Explanatory Functions of Evolutionary Origins
- 3.5 Evolutionary Origins of Aesthetic Themes
- 3.6 Features and Functionalities of Aesthetic Responses
- Part 4 Reinforcing Effects of Aesthetic Phenomena
- 4.1 Reinforcing Effects of Learning
- 4.2 Surprise in Synergetic Interactions
- 4.3 Reinforcing Effects of Concept Learning
- 4.4 Reinforcing Effects of Repertoire Refreshment
- 4.5 Maintaining the Aesthetic Impact of Familiar Works
- 4.6 Why We Continue to Enjoy Great Works
- 4.7 Reinforcing Effects of Power Amplification and Parsimony
- 4.8 Why Parsimony Is Attractive
- 4.9 The Biological Utility of Parsimony
- 4.10 Reinforcing Efffects of Displacement of Aversive Stimuli
- 4.11 Reinforcing Effects of Audience Involvement
- Part 5 The Roles of Mirroring
- 5.1 Mirroring and Imitation
- 5.2 The Biological Utility of Operant Imitation
- 5.3 Emotional Mirroring as an Ingredient of Synergetic Brews
- 5.4 Potentiation by Emotionalizing Feedback
- 5.5 The Biological Utility of Emotional Mirroring
- Part 6 Priming and Potentiating Factors
- 6.1 The Pervasiveness of Priming in Biology
- 6.2 Forms of Priming
- 6.3 The Priming of Paths through the Concept Repertoire
- 6.4 Factors that Potentiate Synergetic Interactions
- 6.5 Culture as a Potentiating Factor
- 6.6 The Diversity of Aesthetic Memes
- Part 7 Devices Used in Creating Aesthetic Effects
- 7.1 Repetition and its Biological Significance
- 7.2 Symmetry
- 7.3 The Use of Symmetry in Synergetic Brews
- 7.4 Descriptions of the Devices
- Part 8 Aesthetic Effects in the Arts
- 8.1 Evidence for the Theory
- 8.2 Use of the Devices in Poetry
- 8.3 Use of the Devices in Literature
- 8.4 Use of the Devices in Music
- 8.5 Use of the Devices in the Visual Arts
- 8.7 Use of the Devices in Performance Art
- 8.8 Use of the Devicesin Architecture
- 8.9 Use of the Devices in Culinary Arts
- 8.10 The Devices in Natural Aesthetic Phenomena
- Part 9 “Aesthetic” Effects in Other Disciplines
- 9.1 Semantic Issues
- 9.2 The Devices in Film
- 9.4 The Devices in Videogames
- 9.5 The Devices in Humor
- 9.6 The Devices in Science
- 9.7 The Devices in Mathematics
- 9.8 The Devices in Chess and Go
- 9.9 The Devices in Actions of Individuals and Groups
- 9.10 It Does Not Matter What We Call It
- Part 10 Future Avenues of Inquiry
- 10.1 Mainly or Purely Mental Synergetic Brews
- 10.2 Methodological Issues
- 10.3 Alternative Research Stretegies
- 10.4 Observations and Conclusions
- 10.5 Final Comments
- Appendix A—Research Topics for Experimental Analysis
- Appendix B—Prior Work on Aesthetics
- References

## The Quest for a Theory

### A Provocative Phenomenon

Beautiful! Wow! Brilliant! Aha! Eureka! Magnificent! Amazing! Awesome! Elegant! Hilarious! Moving!—responses to music, visual art, poetry, film, literature, mathematics, science, performing arts, humor, architecture, culinary arts, and games like chess. What do these reactions, and the events that evoke them, have in common? What evokes them? What are their biological origins?

Although phenomena we may call “aesthetic” permeate our daily lives, there is nonetheless something special about them. This may be why they captured the attention of many of the world’s greatest thinkers through the ages, from the ancient Greeks through today’s neuroscientists. And yet a general and scientifically satisfying theory of aesthetics is still elusive. This article takes an approach that opens aesthetics to the experimental methods of the behavioral and biological sciences, and positions aesthetics as a special case of a pervasive type of human behavior.

#### Why I Took an Empirical-Naturalistic Approach

Mainly, because it keeps the focus on observable phenomena and minimizes bias due to preconceptions, always a potential hazard when the subject matter is culturally entrenched. I wanted to start with an open mind and minimize hypotheses—B.F. Skinner's recommendation for the scientific exploration of uncharted territory (Skinner, 1938). To make sure that any conclusions I might draw would apply to a broad range of disciplines, I examined hundreds of phenomena we tend to call aesthetic, drawn from 17 diverse arts and disciplines, and some from nature. I wanted to discover what, if anything, all of these phenomena have in common.

### What Is Special About the Stimulus?

I observed that stimuli that evoke responses we might call aesthetic are always *composites of multiple elements that don’t ordinarily occur together.* When they do, their joint effect is *different in kind* from the separate effects of the individual elements. *They interact transformatively.*

An iconic example: Picasso combined and transformed two familiar concepts—the handles and seat of a bicycle—into a surprising third concept, the head of a bull. Neither the handles nor the seat would produce this effect separately. Their interaction is thus transformative, and can evoke an “aesthetic” 1 response in viewers who have the relevant priming history with respect to bicycle parts and bulls. A behavioral and biological analysis of aesthetic phenomena requires an examination of the stimulus, the response, the devices responsible for their interactive effects, and the evolutionary origins of these effects.
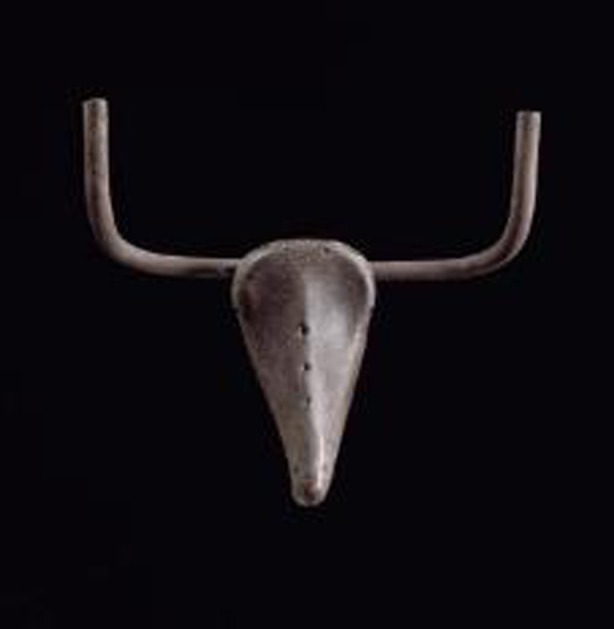


### The Aesthetic Response

In our daily lives, we routinely experience likes and dislikes, small ones and large ones. Our reactions to the mundane stimuli we encounter range from beautiful to ugly, attractive to unattractive, appealing to unappealing. When minor and ephemeral, these reactions usually go unnoticed. When large, they may give rise to the kinds of exclamations listed in the first lines of this article. Intense or fleeting, the aesthetic response proper is of the emotional type, usually covert (private), and often surprise tinged (as in the bicycle/bull example). It may or may not be accompanied by an overt manifestation.

Our private emotional reaction^2^ to a stimulus we find beautiful or surprising, such as the transient pang occasioned by a sunset or a Mozart melody, does not depend on any effects it may have or on what it may accomplish; it is unaffected by its consequences or outcomes, and it is often quasi-reflexive. Neuroscientists have observed that the amygdala, whose function Eric Kandel (2012) characterized as “the orchestration of our emotional life,” is an important locus of neural correlates of aesthetic responses. For highlights of the vast literature on prior work in aesthetics, see Appendix B.

### Operant Components

The covert emotional response is distinct from the operant actions that potentiate it—looking at the painting, listening to the music, reading the poem, or putting food in the mouth. Such potentiating operant^3^ actions are necessary precursors of the emotional component, which is generally covert and private. Though covert, it may give rise to *overt* operant responses, like gasps or exclamations (“Wow! “Beautiful!” etc.). These are easily conflated with their covert antecedents, and when they are, the covert antecedent is easily overlooked.
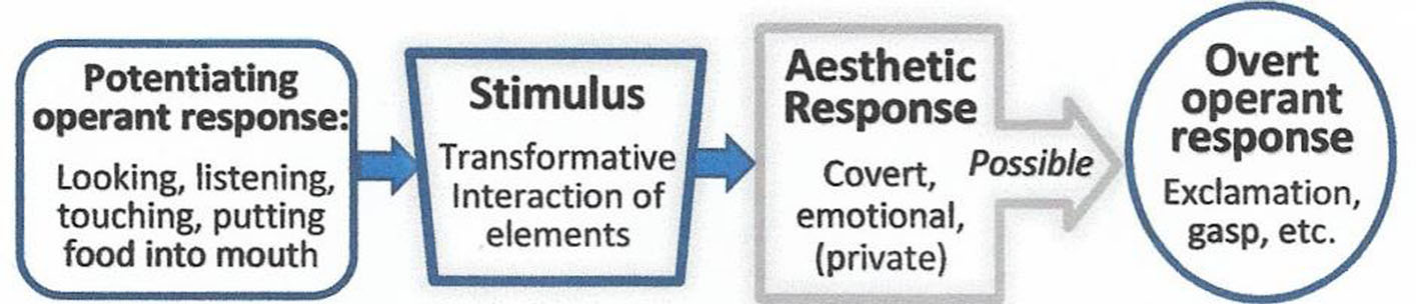


This diagram shows how the aesthetic response is comprised of overt operant and covert emotional components.

But the main reason the covert component is easily overlooked and has not yet received systematic research attention is that it is private, and thus difficult to observe and measure separately (though fMRI technology may be changing that). As such, it is not as accessible for study as, for instance, our verbal behavior.

The aesthetic response is usually surprise tinged (see Section 4.2). It is always modulated by the audience’s^4^ priming history (see Sections 6.1 and 6.2), and by ambient circumstantial factors that potentiate it (see Sections 6.4 - 6.6). It has no immediate function or biological urgency. Part 3 of this article discusses its evolutionary origins.

### Applications Beyond Aesthetics

Aesthetic responses may be special cases of a more widespread behavioral phenomenon—the continuous daylong stream of our fleeting, barely noticed or unnoticed affective reactions to the events and stimuli of our daily lives—the small likes and dislikes, inclinations, and aversions previously described in Section 1.3. Are these subaesthetic or microaesthetic reactions coextensive with aesthetics? The present article identifies many variables that modulate aesthetic reactions. If future research shows these same variables to modulate subaesthetic reactions in similar ways, the conjecture that aesthetic reactions and subaesthetic reactions are indeed coextensive, “made of the same stuff,” would be supported. A fuller understanding of aesthetics might then cast some of its light on such clinical topics as flat affect, despondency, depression, and so forth, and perhaps even on ways to enhance enjoyment of life generally.

### Synergetic Interaction: Nature’s Master Key

The transformative effects described in Section 1.2 were conceptualized by Hermann Haken, the German physicist who originated *synergetics*, the science of interactions whose effects are different in kind from those of the individual interacting elements (Haken, 1978, 2013). He described synergetics, in the title of one of his publications, as *Erfolgsgeheimnisse der Natur*, which translates as “nature’s secrets for achieving successes.” Buckminster Fuller defined synergetics as “behavior of whole systems unpredicted by the behavior of their parts taken separately” (Fuller, 1975), for example, chemical reagents reacting to form other substances; catalytic reactions like photosynthesis; and interactions of DNA’s components to produce proteins and ultimately organisms.

### Synergetic Interactions in Behavior

Given the ubiquity of synergetic interactions in nature, their prevalence in the behavioral realm is not surprising. Familiar behavioral instances (not necessarily pertinent to aesthetics) are learning, trauma, epiphanies, and emotional reactions. For example, the words of a sentence interact synergetically when they are arranged in a certain order—they acquire a meaning that they do not have individually.

Particularly striking instances of behavioral synergetic interaction effects are the extraordinary ones of film and video games. The film medium is unmatched in its ability to capture and hold for hours the rapt attention and intense emotional involvement of an audience. And video games are notorious for their power to exert a virtually addictive hold on the behavior of players, sometimes even at the expense of their social relationships or work obligations.

These behavioral phenomena (again, not necessarily pertinent to aesthetics) involve elements interacting to create effects that differ in kind from the effects of the elements acting separately. The effects always depend on elements occurring together in an unusual combination and interacting transformatively—the definition of synergetic interaction.

### The Synergetic Brew

I will use the term *synergetic brew* as a shorthand for “amalgam of elements, some of which interact synergetically.” Other elements of the brew may interact in other ways—they may be mutually contradictory, incongruous, synergistic, and so forth. The term *brew* encompasses the many different kinds of elements that may comprise it. These may consist of the following:

- any type of stimulus—exteroceptive, internal, or abstract;
- covert (private) effects of verbal or nonverbal thinking (Rolls, 2005);
- any type of emotion;
- social factors and the behavior of others (Malott, 2015, 2016);
- elements of the existing concept repertoire (see Part 2);
- elements generated by audience involvement;
- prevailing behavioral contingencies;
- environmental states like ambient temperature; and
- level of physiological states like hunger, thirst, fatigue, or pain.

Synergetic brews comprised of these types of elements act as stimuli for many types of emotional responses, including aesthetic and subaesthetic ones. The multitude of possible interactions among the elements of synergetic brews—the interactions’ historic frequencies, durations, probabilities, contexts, sensory modalities, and so forth—offers whole new vistas for studying the genesis of emotional reactions.

### Synergetic Effects in the Arts

Elements that interact synergetically normally don't occur together. If and when they do, they interact only when the interaction is appropriately primed and potentiated. The effects of synergetic interactions are termed aesthetic when they generate responses of the types described previously in Sections 1.3 and 1.4.

Artists, composers, poets, and others arrange the elements of their disciplines to create synergetic brews that have aesthetic effects for appropriately primed audiences. These brews bring together stimuli that may be unremarkable individually for "a magical metamorphosis of the ordinary" (Gibson, 2011). For example, the interacting elements may include a particular melody, rhythm, and harmonic progression, or a certain color scheme, composition, and depiction. Synergetic interactions often occur when the interacting elements are drawn from diverse disciplines. For example, musical and verbal concepts may interact synergetically to create songs; the emotional impact of film is often the result of interactions among music, color, drama, and emotional concepts; the dramatic impact of an operatic work may be due to synergetic brews that combine music, plot, and staging; a cathedral’s architecture and singing can enhance the congregation’s religious fervor; and in paintings, emotion-evoking subjects like violence, nudity, religious concepts, or emotional facial expressions can interact synergetically with the theme, color, or composition.

### Devices That Create Effective Synergetic Brews

A remarkably small set of concept manipulation devices (I have identified 16) accounted for all of the hundreds of observed effects. These devices are summarized in Section 7.4. All of them are based on manipulation of the audiences’ concept repertoires and they reflect the ways in which the elements of the brew interact. The devices can be thought of as recipes for the creation of synergetic brews that can then be configured into Works.^5^

The operation of these 16 devices is responsible for the aesthetic emotional impact that some synergetic brews create. Parts 8 and 9 provide more than 200 examples of how they create synergetic brews. For each of the effects discussed, I referenced the specific devices at play with the letters under which they are described in Section 7.4.

### Potentiation and Priming of Aesthetic Responses

Responses to synergetic brews occur when they are potentiated. While chemical or culinary brews often require heat or catalysts for their potentiation, synergetic brews that evoke aesthetic responses require potentiating factors that include the audience’s behavioral and physiological state as well as environmental and sociocultural factors (see Sections 6.4 - 6.6). Response-produced stimuli, too, can potentiate and stoke the synergetic interaction via autocatalytic feedback.

The audience’s behavioral state must always include the effects of an appropriate priming history with respect to the brew’s ingredients (see Sections 6.1 and 6.2) and the right emotional or physiological state. Fear, anger, hunger, cold, pain, for example, generally inhibit aesthetic as well as subaesthetic responses. Audience members respond to synergetic brews idiosyncratically due to the uniqueness of their concept repertoires (see Section 2.3) and priming histories.

### Common Features of Aesthetic Phenomena

Per our theory, the five features listed below, taken together, describe the main attributes of aesthetic phenomena as the term is generally used in most verbal communities.

- An audience's response to synergetically interacting elements.
- A response that has a surprise-tinged emotional component.
- A synergetic brew’s positive reinforcement effect.
- An audience in an appropriately primed state (see Sections 6.1 and 6.2).
- Factors that potentiate the response (see Sections 6.4 - 6.6)

The synergetic stimulus is always a complex of the types of elements listed in Section 1.7, and the aesthetic response itself is described in Sections 1.3 and 1.4.

### A Diagrammatic Overview

The following figure presents my metaphors for the creation of aesthetic responses. The sketches at the upper right represent creators of aesthetic effects as they combine and configure the elements of their arts. Some of these elements are listed in the broad arrows that lead to the cauldron. The devices the creators use in creating the synergetic brews are represented by the manual, *Devices That Can Create Aesthetic Impact*—their conceptual “cookbook.” The cauldron represents the disciplines (art, music, etc.) in which the creators configure synergetic brews into Works. They apply creative energy symbolized by the flames under the cauldron. The Works become accessible to audiences via potentiating factors symbolized by the ladle and dish. For audiences (the face) that have the requisite priming history, the Works evoke covert emotional aesthetic responses sometimes accompanied by overt manifestations, like the exclamations shown in the bubble on the left. The smile is intended to indicate that synergetic stimuli that evoke aesthetic responses are emotionalizing and often positively reinforcing.
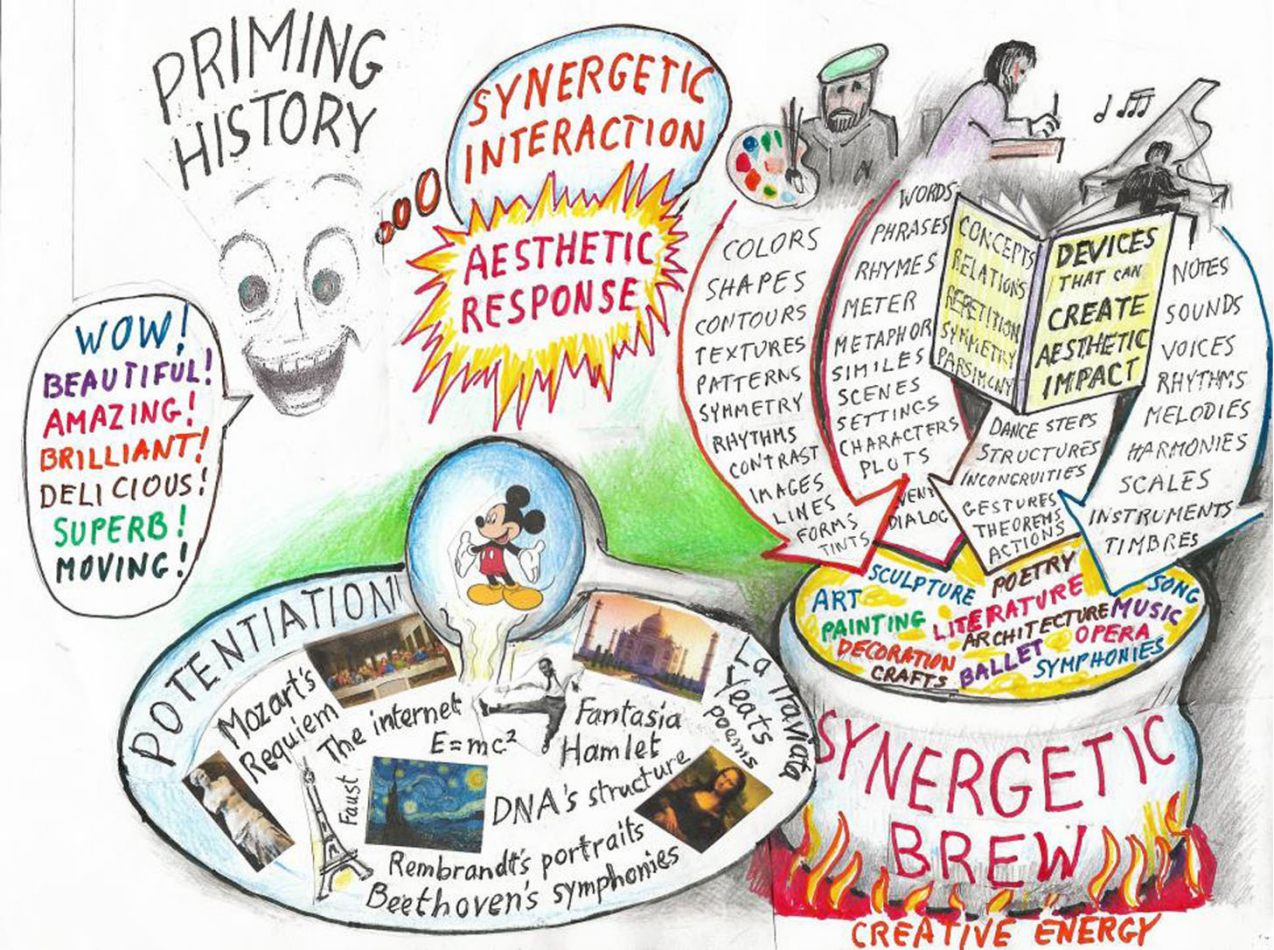


### Synergistic Versus Synergetic Interactions

As mentioned in Section 1.8, not all elements of a brew interact synergetically. Many may interact only *synergistically*, or not at all. *Synergetic* interactions differ from *synergistic* ones in that their effects are transformative (different in kind from the interacting elements), whereas those of *synergistic* interactions are quantitative and incremental (greater than their sum, as if 1 + 1 = 3), but not different in kind. Both synergistic and synergetic effects can be involved in the creation of aesthetic effects in synergetic brews.

This figure illustrates the difference between synergetic and synergistic interactions of elements. The analysis of such interactions is still unexplored territory in behavioral science. The challenge is to analyze, describe, and categorize interactions on the basis of their features and functions. For instance, synergistic interactions among elements often create new elements that will then interact synergetically (transformatively) with other elements. Thus, synergistic interactions can potentiate synergetic ones. The study of types of interactions may prove to be a fruitful area of research (see also Section 1.8).
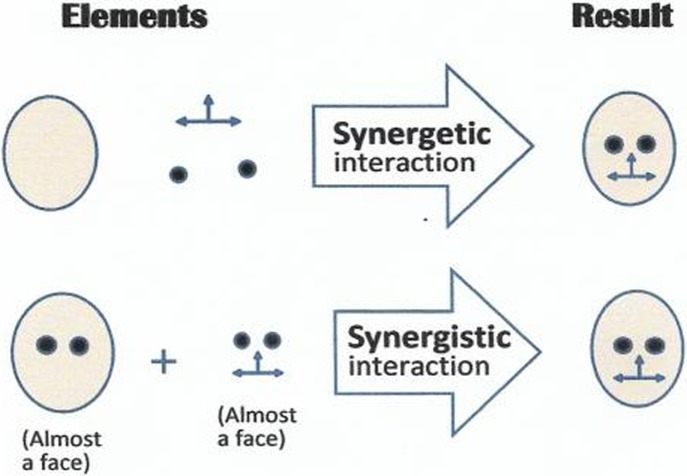


### How the Present Approach Differs

It comes from a special direction—the empirical-naturalistic direction—and uses special analytic tools. I tried to confine myself to concepts that apply to observable entities, or that can be modeled for experimental analysis, at least in principle (see Section 1.17).

Parts 8 and 9 present more than 200 of the many “aesthetic” effects I analyzed. To help ensure that any commonalities I may observe are general, I gleaned the samples from 17 different arts and disciplines and also included some natural aesthetic phenomena (like sunsets).

The preceding sections presented observations regarding the nature of the aesthetic response and how it is evoked. This is the foundation on which the rest of the theory is built and on which we can address such questions as:

- What attributes of aesthetic phenomena are responsible for their reinforcing properties?
- Are there different kinds of “aesthetic” effects? If so, what are they?
- Does what we learn about aesthetic phenomena apply to subaesthetic ones (as proposed in Section 1.5)?
- Is there an identifiable set of devices that creators of aesthetic effects use, and if so, what are these and why do they work?
- What are the evolutionary roots of aesthetic phenomena?

### The Domain of Emotional Reactions

Most prior work in aesthetics considered mainly phenomena termed beautiful or pleasurable rather than ugly or unpleasant. But the present analysis, as well as some prior ones (e.g., Rolls, 2011; Rusch & Voland, 2013), suggest that all aesthetic phenomena, regardless of valence, involve similar mechanisms and origins. *Beautiful* is to aesthetic phenomena as *sweet* is to tastes (which can also be bitter or sour).

But regardless of valence, synergetic brews that evoke aesthetic responses are usually reinforcing to the audience: even sad or tragic stories attract audiences, not to speak of horror movies and thrillers. The evolutionary theory put forward in Part 3 helps explain why the properties of aesthetic phenomena transcend valence. Part 4 identifies and analyzes the underlying mechanisms and evolutionary roots of their reinforcing and emotionalizing effects.

### Research Models of Aesthetic Phenomena

The main challenge of any research endeavor is to devise simple, manageable laboratory models that lend themselves to experimental analysis and measurement (Nagel, 1945a, b, 1961). The experimental study of aesthetic phenomena will require a variety of such models for the various devices described in Section 7.4. The present theory opens several paths to laboratory modeling of aesthetic phenomena.

For instance, a rudimentary model of expectation and surprise can be created by installing a learning history in which Concept A was always followed by either Concepts B or C*,* whose relative historic frequencies would be an independent variable. Various possible dependent variables can then be used to study the differences between the responses evoked by Concepts B and C.

The film medium and video games warrant special research attention because of their outlier status with respect to both (a) their uniquely powerful behavioral effects and (b) the unusually large number of elements and devices that comprise their synergetic brews (see Sections 9.2 and 9.3). If research were to show (a) to be a function of (b), new light would be shed on the mechanisms of reinforcement and emotionalization in other arts and disciplines. Such a relationship would help identify attributes that synergetic brews must have in order to evoke certain types of responses.

Appendix A outlines the many research topics and directions that the present theory suggests. That outline should be regarded only as a preliminary sketch that needs, and I hope will receive, elaboration as actual research gets underway.

### Research Implications for Subaesthetic Effects

If the same variables that modulate aesthetic responses are shown to have similar modulating effects on the subaesthetic responses described in Section 1.5, we would have further evidence that subaesthetic responses are indeed coextensive with aesthetic phenomena—weaker, subtler, less noticeable, and more ephemeral versions of them. These variables could then be considered research targets with implications even beyond the clinical ones—perhaps factors that impact the normal enjoyment of daily life.

## The Central Concept of Concept 6

### How the Term Is Used

To describe the synergetic brews and devices that evoke aesthetic responses in terms consistent within an empirically grounded conceptual framework, we need the concepts described in the sections that follow. To be useful for a systematic experimental analysis, such concepts must be framed in operational terms and refer to entities that are observable, at least in principle (e.g., Nagel, 1945a, b).

All animals, humans included, continuously discriminate between, and generalize within, classes, and when they do, we say that they behave conceptually (Hull, 1920; Kandel, 2012, pp. 306–307; Keller & Schoenfeld, 1950; Mechner, 1961, 1962, 1967; Skinner, 1938, 1953; Smoke, 1932; Zadeh, 1965). All higher animals navigate their worlds via discrimination and generalization. For instance, they discriminate between categories of edible and inedible things and generalize within each of these categories. Our concept *animal* involves generalizing *within* each animal species, discriminating *between* species and discriminating the class animal from other classes, like plants or inanimate objects.

This conceptualization of *concept* has universal applicability, ranging from the most concrete concepts (e.g., apple, dog, red) to the most abstract or fuzzy ones (e.g., beauty, relationship, space). The notion has foundations in the past century’s body of laboratory research relating to discrimination between, and generalization within, classes. It is also flexible: any instance of a concept is described by a subset of the general concept’s attributes. For a subset of attributes to be sufficient for a valid description of a concept, it is sufficient for the subset to consist of only some of the attributes of the general concept, with no single attribute being necessary.

### Nonverbal Noncognitive Concepts

The above conceptualization of “concept” includes noncognitive and nonverbal ones and is thus broader than the colloquial one, which tends to emphasize verbal and cognitive concepts, often tagged with their category names. The present conceptualization goes beyond the operant and cognitive ones to include emotional responses and the responses involved in classical conditioning. For instance, the salivary reflex generalizes among some stimuli and discriminates these from others. Like operant responses, emotional responses occur selectively, in response to some stimuli and not others (they discriminate between and generalize within classes of stimuli.

Our analysis of aesthetics requires this broad conceptualization of “concept”^7^ and opens the door widely to all methodological options. We may eventually learn that the behavioral architectures of aesthetic responses and their covert emotional components are just as elaborate and conceptually nuanced as those of verbal behavior and language.

### The Concept Repertoire

The millions of concepts that make up our concept repertoires include visual ones, like colors and shapes; auditory ones, like melodies and voices; tactile ones, like textures and sensations; abstract ones, like mathematical relations; actions of people; and myriad social, emotional, and verbal concepts. Concepts may be operant or respondent, verbal or nonverbal, cognitive or emotional.

Every concept in our repertoires, from the simplest to the most complex and from the most concrete to the most abstract, is currently or potentially related to millions of other concepts by millions of possible relations. Those relations can be temporal, sequential, spatial, equivalent, logical, intensive, and so forth. And every relation is itself a concept, a member of a class of relations, thereby creating a vast relational framework (Barnes, 1994; Hayes et al. 2001).

Our concept repertoires change from instant to instant due to interaction with the environment and covert activity, like thinking. Concepts and their relations may be current (already part of the repertoire) or potential. Potential relations become current when they are perceived, learned, or realized. When a multiple-link chain of potential relations becomes a single-link current relation (Fields & Moss, 2008; Fields & Verhave, 1987), the chain is, in effect, short-circuited (see also Section 6.3). We will examine how the conversion of potential relations into current ones can have an aesthetic impact.

### Concepts and Their Relations

The “strength” of a concept in an individual’s concept repertoire depends mainly on the number of other concepts to which it is related, and *their* strengths. The stronger a concept, the more easily it forms relations with other concepts—the rich get richer.

Arntzen (2004; Arntzen, Nartey, & Fields, 2015) showed that multiple-link chains of equivalence relations form more readily when one of the linked concepts is a strong and “meaningful” one, like a picture of a familiar object, than when all the concepts are weak (abstract or have few relations). Thus, strong concepts function like beacons that light up paths through the concept space.

A concept’s degree of functionality depends on its state of priming. When the concept is not fully functional, a small amount of additional priming may carry it over the functionality threshold (see also Sections 6.1 - 6.3)—a synergetic effect.

### Why Concepts Are Central to the Theory

All of the aesthetic effects I examined—many hundreds, including the more than 200 presented in Parts 8 and 9—involve devices based on the manipulation of concepts—formation of new ones, reconfiguration or displacement of existing ones, expansion of their classes, and/or increase in the number of their relations. Parts 2 - 7 explain how the devices described in Section 7.4 achieve these types of concept manipulation functions, and Parts 8 and 9 provide examples of how those devices participate in creating the described effects.

## Evolutionary Roots of Aesthetic Phenomena

### Emotion-Evoking Effects of Synergetic Brews

All of the hundreds of aesthetic responses I examined involved synergetic effects of interacting elements, where at least one of the elements was *not ordinarily present.* The conjunction of some of the elements was always unusual in some way. But these observations apply to *all* emotional reactions, including nonaesthetic ones, and all are evoked by synergetic brews.

What, then, distinguishes the synergetic brews that evoke *aesthetic* reactions from those that evoke the myriad other types of emotional reactions? To answer this question, it is necessary to consider the biological functions and origins of emotional reactions during our evolution.

Emotional reactions evolved as rapid responses to emergencies, like an imminent threat that must be avoided or a sudden opportunity that must be seized. Regardless of the urgency of the required action, the alarm signal itself may be subtle. In our primordial environments, a minor discrepancy—such as a change in a pattern, the snapping of a twig, or an unfamiliar object—can signal a serious emergency. Even mundane and familiar stimuli can be alerting and attention getting when they occur in a context that gives them special significance. A disposition to notice unusual events or deviations from usual patterns would thus have been selected. The silhouette of a hawk in the overhead sky is alarming to chicks, and a darting toy mouse is exciting to a kitten.

### A Theory of How Aesthetic Responses Evolved

Given that the tendency to react emotionally to biologically significant stimuli, like threats or opportunities, was selected during our evolution, might this tendency be the precursor of aesthetic responses?

A theory: During the course of our evolution, synergetic stimuli that signaled alerts gradually expanded the range of their functions to less urgent ones and eventually to ones that had no urgency at all and required no action. Accordingly, the character of the responses also changed. Their urgency removed, the emotional component became subtler and more nuanced—often surprising and/or informative rather than alarm driven. The end-point of this process--synergetic brews that evoke aesthetic responses—*do not call for any action at all and have no urgency*.

#### Fusion With the Procreational Fitness Theory

Another evolutionary pathway, one that has often been proposed (see Appendix B, the paragraph titled Conjectures Regarding Evolutionary Origins), is that of primordial mating displays (songs, dances, and various types of antics) that serve as an advertising medium for the performer’s procreational fitness.

Like the alarm theory, this theory, too, implies a progressive diminution and eventual disappearance of urgency. Mate selection decisions in response to mating behavior may originally have had urgency and biological significance, but today’s arts have few if any. The evolution of aesthetics may thus have included a merger of the alarm theory and the procreational fitness theory.

### Evolution of the Beauty Concept

The mating-display theory may have been one of the pathways for the evolution of the pervasive concept of beauty. In species that select reproduction partners on the basis of their performance in mating displays, a reproductive advantage would have accrued to those with the disposition and discernment to be attracted by competently executed (“beautiful”) performances.

Though the mate selection functionality of displays and performances may largely have fallen away, the disposition to be attracted by the various features described in the sections on symmetry (Sections 7.2 and 7.3), parsimony (Sections 4.7 - 4.9), and the various other devices described in Section 7.4 would have been preserved and may have evolved into our present-day aesthetic sensibilities, in accordance with the pathways proposed in Section 4.9 for parsimony and Section 7.3 for symmetry.

### The Explanatory Functions of Evolutionary Origins

The theory that aesthetics has its evolutionary roots in the gradual broadening of the alerting function and the procreational fitness advertising function does more than fill an important explanatory gap: it is also a source of prompts for identifying various features of aesthetic phenomena that might otherwise escape notice and for explaining the functionality of those features, including their positively reinforcing properties. Among these features are symmetry, parsimony, concept learning, surprise, and repetition.

### Evolutionary Origins of Aesthetic Themes

An account of the evolution of aesthetics provides hints for where to look as we try to parse its nuances and subtleties. Tracing these to their pre-aesthetic roots often points to significant present-day features that may otherwise escape notice. Here are some examples:

*Dance—*Of all the art forms, dance, with its physical and erotic attributes, is the one most readily traceable to primordial mating displays and performances (see also Section 8.7).

*Romance*—Many of the devices described in Section 7.4 make use of such emotion-inducing themes as love, jealousy, fidelity, betrayal, rivalry, scheming, and familial and tribal dynamics. All of these are easily traced to primordial evolutionary roots that involve emotionalizing situations of danger or opportunity requiring urgent action.

*Danger—*Themes related to danger, threat, escape/avoidance, or suspense, widely used in film, literature (thrillers), and all kinds of games, are obviously and directly related to the alarm and danger signals in their evolutionary roots.

*Voice—*For most terrestrial animals, voice is the main medium for signaling danger, opportunity, or calls for urgent action. It is therefore not surprising that voice has evolved into one of humankind’s most powerful media for the conveyance of emotion, as via vocal music, speech, poetry, and oratory.

*Competition—*Artists, performers, and other creators of aesthetic effects commonly have a strong tendency to compete for approval and acceptance of their works, as well as for personal status and fame, to a degree not readily explained by economic factors. The procreational fitness theory of the evolutionary roots may help explain this tendency.

*Humor—*Since humor is based on the identification or creation of incongruities, contradictions, or double meanings (see Section 9.5), it often functions as a medium for displaying the humorist’s conceptual facility and discernment regarding conceptual relations. This function suggests roots in the primordial advertisement of procreational fitness via displays of proficiency.

### Features and Functionalities of Aesthetic Responses

When evolution generates a new variant of a biological feature (e. g., scales evolving into feathers), that variant may find new functions. As it becomes elaborated over time, it loses some of its original features, retains some, and adds some new ones.

#### The Retained Features of aesthetic phenomena

- The synergetic brew’s attention-getting feature—a potentiating factor that the aesthetic response still requires for its evocation
- The synergetic brew’s emotion-evoking effect
- The requirement that at least one of the synergetic brew’s elements is unusual, that is, not ordinarily present, evokes the surprise-tinged emotional component
- An advertising medium for the general competence of creators of aesthetic effects, though no longer for procreational fitness
- Susceptibility to enhancement by the 16 devices described in Section 7.4.

#### New Features and Functions of Aesthetic Phenomena

These are a consequence of the newly evolved nuances of the elaborated emotional responses to the widened range of synergetic phenomena that the ancient Greeks termed *aisthētikós*.

The main new functionalities:

- Expanding the audience’s repertoire of concepts and conceptual relations (Part 2)
- A new type of positive reinforcement: the pleasurable effects of synergetic brews that evoke aesthetic responses (Part 4)
- A rich communication medium
- A glue for social cohesion and a medium for meme formation, similar to language (Section 6.5)
- A medium for emotional stabilization and displacement of aversive states (Section 9.5)

As our evolutionary ancestors made contact with these new features and functions, they learned to control and enhance them and developed various devices for doing so (see Sections 1.10 and 7.4). They learned to use these devices to increase the reinforcing effects of those features. This may be how the arts developed.

#### Lost Features and Functions of the Evolutionary Antecedent

- Requirement for a specific active response
- Urgency of the response
- Sensitivity of the response to consequences
- The alarm function

## Reinforcing Effects of Aesthetic Phenomena

A synergetic brew that gives rise to an aesthetic response is usually also a positive reinforcer, even when some of the elements are saddening, frightening, or horrifying. The reinforcing effects do not fall into any of the traditional categories like alimentation or sex and do not have separate names. And yet they are distinctive and can be powerful.

The sections that follow explain some of the origins of these special types of positive reinforcement effects. They fall into five broad categories:

- instructional
- refreshment of existing concepts and relations
- effects involving power amplification/parsimony
- displacement of aversive emotional states
- effects generated by active audience involvement

These same types of reinforcing effects are also applicable to the far more pervasive subaesthetic responses discussed in Section 1.5.

### Reinforcing Effects of Learning

Instructional episodes increase the learner’s ability to predict and control the environment. Ability to do so has biological utility, which is how learning experiences acquired positively reinforcing properties during our evolution (Rolls, 2005) and how it came about that curiosity, exploratory behavior, and the tendency to find novel stimuli reinforcing became widespread in the animal kingdom. It may also explain why puzzle solving is so highly reinforcing to many people (e.g., crossword and jigsaw puzzles, solitaire, video games.)

The film medium may owe some of its unique reinforcing power to its ability to provide a wide variety of learning experiences to its audience, and to do so with a high flow rate. Prominent among these are realistic renderings of novel environments, both natural and human; exposure to instructive interactions; vicarious survival of dangers^8^; and observing others navigating their environments and solving problems.

The other major reinforcing effect that both film and video games offer is that of simulated or vicarious power amplification, discussed in Sections 4.7 - 4.9.

### Surprise in Synergetic Interactions

Since our vocabulary does not come close to providing names for all the nuanced emotional responses that are evoked in the arts and sciences, I am using the term *surprise* as a stand-in for the entire wide and nuanced range encompassing pleasure and exhilaration; fear or sadness as in tragedy (Aristotle’s *Poetics*, 335 BC); tears as in Walt Disney’s classic children’s movies; horror as in thrillers; anger; laughter; and other emotional responses.

The term *surprise* commonly refers to the covert emotional reaction when an expectation is not realized, an unexpected event occurs, or new information is received. The surprise may be subtle or blatant, and may or may not give rise to an overt manifestation: if subtle, perhaps a smile; if major, perhaps “Wow!”

#### The Biological Utility of Surprise

Neutral or innocuous stimuli can interact synergetically to signal surprising or informative events, like opportunities or dangers. Unremarkable, minor discrepancies can assume new significance when they occur together in certain contexts. A tendency to notice strange sounds or unfamiliar objects—usually meaningless but potentially a matter of life-and-death—undoubtedly had survival value during our evolution. A footprint in the sand evoked a strong emotional reaction from Robinson Crusoe when it interacted synergetically with his belief that he was the island’s only inhabitant, in conjunction with his recognition that the footprint was not his own.

A common type of surprise event is the non-confirmation of an expectation.^9^ We generally learn more when an expectation is not confirmed than when it is. When surprising events are thus viewed as learning experiences, their biological utility is evident. The same applies to the *confirmation* of an expectation, which can be instructive (and thus reinforcing) when it resolves an uncertainty.

### Reinforcing Effects of Concept Learning

A concept’s relations to other concepts correspond to what is often referred to as its “meaning” (Fields & Arntzen, submitted). Adding a new relation to a concept thus amplifies its meaning. A familiar example is the mutual amplification of meaning between a melody and its lyrics, as in songs. The meaning of either would be very different in isolation. Another familiar example is the addition of a soundtrack of “scary” music to danger scenes in thriller movies. All of these emotion-inducing effects are instances of synergistic or synergetic interactions.

When a new relation between two concepts is learned, it often transfers to other relations automatically. For instance, Concept A is the price of one item, Concept B is the price of *n* such items, and the relational concept is multiplication by *n*. Once learned, this relation may transfer to other situations where the measure of one unit is known and that of *n* such units is sought. In general, establishing a new relation often generalizes to similar situations and concepts (Mackay & Fields, 2009), with an expansive effect on that concept's class and hence on its biological utility and reinforcing effect.

#### Class Expansion Devices

The term *class expansion* refers to the increase in the members of a class among which one generalizes when behaving in accordance with that concept. For example, a coral belongs to the class of animals. Many of the devices described in Section 7.4 involve class expansion and the formation of new relations.

### Reinforcing Effects of Repertoire Refreshment

We normally find it reinforcing to exercise our capabilities, perceptual and motor. We go for walks, look around, listen to sounds, handle objects, touch things, savor foods, socialize, and exercise our bodies. Such activities are biologically useful because they refresh and maintain our physical and behavioral functions. In the case of our concept repertoires, *they refresh and maintain our discriminations and generalizations*. Like most biological functions, if we do not use them, they deteriorate: muscles get flabby and concepts get blurry. That is how we evolved to find refreshment and maintenance activities reinforcing.

Certain behaviors can be refreshed only when they occur. To refresh our concept of a melody or voice, we need to hear it again, or of a painting’s color scheme or of a face, to see it again. We can refresh our ability to predict what comes next in a sequence only via exposure to the precursor, as when we listen to music or reread a poem.

### Maintaining the Aesthetic Impact of Familiar Works

What does it mean to have become familiar with a piece of music, a painting, a poem, or any other work? It means having learned some of its constituent concepts—musical, coloristic, phonological, or abstract—and the relations among them. We are familiar with a work when some of its features have become part of our concept repertoire. Again, it is important to keep in mind that such concepts are not necessarily verbal or cognitive—they can be purely visual, purely auditory, purely abstract, or various combinations of these.

When we listen to music, our moment-to-moment expectations as to what comes next are continuously confirmed and disconfirmed. This also happens as the eye moves across a familiar painting or as we read a familiar poem. Those exposures refresh, update, and maintain the concepts involved. One reason for our enjoyment of familiar works is the reinforcing effect of refreshment.

### Why We Continue to Enjoy Great Works

Nonetheless, repetitive exposures to any work diminish the levels of the mini-surprises it holds, mainly because of habituation or extinction. So why do we still enjoy listening to a recording over and over, or keep looking at the same painting, even long after the original surprise effects have worn off, with the aesthetic impact of the synergetic interactions correspondingly diminished?

Explanation: Each successive exposure to a work alters our response to it and thus impacts a somewhat altered concept repertoire. Even though the musical recording and the painting do not change from one exposure to the next, we do. Our concept repertoires change as a function of successive exposures to the work, the passage of time, intervening events, and neural activity. Thus, the next exposure to the work reinstalls its concepts in the altered concept repertoire environment. The resulting refreshment effect is often sufficient to override any habituation-caused loss of aesthetic impact.

This may also explain why an immediate repetition of the same song or poem is rarely as pleasurable as hearing it again after a longer time. The longer the time, the greater the need for refreshment of the concepts involved and the more reinforcing the next exposure is likely to be.

### Reinforcing Effects of Power Amplification and Parsimony

Why is parsimony—economy of means or effort—generally pleasing, and why is it often an ingredient of the synergetic brews that contribute to aesthetic effects? The answer may reside in the close relationship between parsimony and power amplification.

Examples of power amplification are action at a distance like throwing, shooting, slinging, or use of the lever principle—all of which are instances of large physical effects produced with comparatively small input effort.^10^ Other examples are the achievement of multiple effects with single acts, as when issuing a command to many individuals, broadcasting, or making long-lasting marks as in stone engravings, sculpture, and graffiti. It is easy to see how such power amplification would have been biologically useful during our evolution and would thus have acquired reinforcing properties.

### Why Parsimony Is Attractive

But how is power amplification related to parsimony? As human behavior repertoires came to include language and abstract concepts, the reinforcing properties of economy of means and effort (power amplification) *generalized beyond physical power to explanatory and predictive power—*to abstract domains like verbally formulated relationships among concepts, as in logic or mathematics. In accordance with Ockham’s razor, we characterize a statement as parsimonious, simple, or elegant when a small number of words or symbols predicts or describes a large number of phenomena (see Sections 9.6 and 9.7).

The behavioral scientist’s challenge is to explain how it came about that parsimony is considered desirable—beautiful, pleasing, elegant, positively reinforcing. Often, a good heuristic is the phenomenon’s biological utility, like its relevance to our evolutionary ancestors’ ability to predict and control their environments.

“Beauty is truth, and truth beauty,” wrote John Keats in *Ode on a Grecian Urn*. Mathematicians and logicians agree when they interpret truth to mean formal consistency. Scientists agree when they interpret truth to mean empirical validity and correctness: “Too beautiful to be false,” is the way Einstein referred to his general theory of relativity (Marr, 2003), and James Watson made a similar comment regarding the double helix theory (Mukherjee, 2016; Watson, 1968). Thus Einstein, Watson, Keats, as well as countless scientists, mathematicians, and philosophers through the ages have tacitly equated parsimony with beauty, truth, and empirical correctness.

### The Biological Utility of Parsimony

A possible explanation, in terms of biological utility, invokes the observed correlations between parsimony, prediction, and control. Einstein and Watson viewed these correlations as so self-evident and supportive of the validity of their theories that no further explanation was required. But the behavioral scientist requires one.

The main explanation is that order allows for more and better prediction and control than chaos. Order is associated with simplicity, and simplicity with parsimony. To the extent that our evolutionary ancestors made contact with these relationships as they led their lives and interacted with their environments, parsimony would have been expected to acquire positively reinforcing properties, variously termed beautiful, pleasing, simple, or elegant. Thus, perceiving parsimony as beautiful and desirable would have become a selected characteristic during our evolution.

### Reinforcement Effects of Displacement of Aversive Emotions

Stimuli that evoke smiles or laughter are usually positively reinforcing. Depending on the genre of the humor, they may be called humorous, funny, witty, or amusing. The distinguishing features of humor are discussed in Section 9.5.

One mechanism for the reinforcing effect of humor is its ability to displace aversive emotions, like fear, anxiety, disappointment, discomfort, frustration, anger, sadness, or gloom (e.g., gallows humor). Humor often achieves such displacement by generating competing incompatible emotions.

Another mechanism involves concept manipulation and learning. Since humor is always based on the identification of incongruities, contradictions, or double meanings, the effect is to reveal hidden relations or other unexpected kinds of class expansion.

Humor is also a medium used by its agent to display facility, proficiency, and discernment in identifying conceptual relations and using them. Given humor’s reinforcing properties and its consequent power to entertain, the agent gleans the audience’s appreciation and approval. The relationship of these dynamics to their evolutionary roots in the procreational fitness display theory is evident.

### Reinforcing Effects of Audience Involvement

In certain instances of aesthetic phenomena, the audience’s own behavior contributes to the elements of the synergetic brew. In music, this can involve performing the music, either solo or with a group; in visual arts, copying the work; in poetry, reciting it; in literature, reading it aloud or performing the lines of a play; in performance arts, mirroring the performer (Part 5); in video games, playing them.

Active participation that consists of overt operant behavior adds multiple elements to the synergetic brew—kinesthetic, sensory, and cognitive ones. According to the hypothesis put forward in Section 9.3, adding elements to a synergetic brew may increase its impact. In addition to adding elements to the brew, active involvement also enhances an audience’s perceptions of other elements of the brew, especially ones that may escape notice when the audience is passive.

The evolutionary roots of aesthetics proposed in Part 3 suggest that the primordial danger/opportunity signals, as well as displays of procreational fitness, required active overt responses. Given those roots, it is not surprising that this active response feature survived as a potentiating attribute.

An additional contributor to the reinforcing effect of audience involvement is the creative aspect. While an analysis of the nature of the reinforcement that accrues to the creators of aesthetic effects is beyond the scope of this article, it is likely that audience participation, with its attendant contribution to the synergetic brew, generates some of the reinforcement effects of creative acts.

## The Roles of Mirroring

### Mirroring and Imitation

Monkeys have neurons in their cortex that fire when the monkey performs a certain action, and also when it observes another monkey performing that same action, without the observing monkey actually making the movements (Rizzolatti & Craighero, 2004). Humans have those neurons too. Mirror neurons, as they are called, are involved in overt as well as covert imitation. To mirror someone’s behavior is to perform it at a covert level, mentally, or to “imagine it.” In much of performance learning, as in sports and arts, the learner matches recalled and imagined stimuli (Mechner, 1992). Mirroring can evidently assist learning by imitation, even in the absence of immediate overt follow-up imitative activity: When a learner observes and mirrors an action and retains it in memory, the action can be imitated and matched later, in more convenient circumstances rather than immediately.

### The Biological Utility of Operant Imitation

In addition to the behavior-shaping effects of consequences, imitation is one of the main ways higher animal species learn. Imitation can also have survival value for the group, as when uniformity of action is required. The leader acts, and the herd (or pack, flock, school, bevy, etc., depending on species) follows with automaticity and immediacy. If each member of the group made separate decisions in such situations, the benefits of prompt joint action would be lost. This “follow-the-leader” imitative pattern can have survival value for humans too, as in military or emergency situations.

Imitative behavior can also serve as elements of synergetic brews that have aesthetic impact, for example, accurate mimicry, acts executed in unison, ballets, marching parades, and aquatic displays by cetaceans. Imitation also occurs in situations that are not necessarily beneficial to the larger society, even repugnant, as in certain types of mob behavior.

### Emotional Mirroring as an Ingredient of Synergetic Brews

Covert mirroring of emotions is believed to be at play in empathy, identification, and emotional contagion, as when we resonate with the emotions of others (Iacoboni, 2008). When we imitate or feign anger or laughter in operant fashion, the physiological concomitants of those emotions may soon follow. Professional actors uniformly report that when they act out a character’s emotions they come to experience them, thereby making the resulting performance more convincing.

How is it that performance of an emotion’s operant manifestations often evokes the emotion’s physiological concomitants? A plausible explanation is that the multiple instances in which the emotional response’s operant components were paired with its physiological components, over a lifetime, amounted to classical (Pavlovian) conditioning, with the operant component functioning as the conditioned stimulus.

Emotional mirroring may cause us to resonate with the emotions of a character in a movie or novel, or in a Verdi opera or a Rembrandt portrait. This may also be the reason why live performances are more impactful emotionally than recorded ones that lack the visual element.

### Potentiation by Emotionalizing Feedback

The output of a synergetic interaction can flow back to the input to function as an additional ingredient of the synergetic brew. Such autocatalytic feedback loops are common in biological processes. Familiar examples are metabolic, digestive, sexual, emotional, and other behavioral processes whose outputs augment their own momentum.

Sections 4.2 and 4.3 focused on surprise as one of the emotional components of the response to the synergetic brew—an output component. But surprise can also function as one of the synergetic brew’s interacting ingredients, an input component that may itself be surprising or emotionalizing, potentially in a recursively nested cascade.

In general, synergetic brews are more impactful when one of their ingredients is an emotionalizing one, or when the audience is already somewhat emotionalized. Thus, when the synergetic interaction’s response output is fed back to the synergetic brew, it can help potentiate the response autocatalytically.

#### Mirroring of Emotional Displays

Familiar examples of the potentiating effects of emotion are displays of anger or passion by orators. By shouting or gesticulating, they may induce an emotional state in their audience via emotional mirroring, thereby adding an emotional component to the synergetic brews that include their messages. Such emotional enrichment makes the synergetic brew as a whole more effective. For more on that, see Section 8.7 on performance arts.

Emotional mirroring is also involved in the emotional contagion that occurs in rallies and mob-action situations. But emotional mirroring is by no means the only way emotional states are induced in audiences. Other examples are emotion-inducing background music in the soundtracks of films; the use of emotionalizing subjects in paintings; patriotic background music for nationalist propaganda; and lullabies to relax children. In general, when the emotional component of the response to a synergetic brew feeds back an emotionalizing ingredient, it may augment the response.

### The Biological Utility of Emotional Mirroring

To understand the biological utility and evolutionary origins of emotional mirroring, one must look to its benefits to the group, not just to the individual. For instance, emotional mirroring is an important ingredient of the cohesive glue that binds the members of human societies.

Another important type of utility to the group is an increase in the long-term memorability and recognizability of certain events that have survival implications for the group. These include emotionally charged and biologically significant events like courtship, sex, or danger. When these types of events become tagged with emotion-linked markers like odors, tastes, voices, songs, or images, their recognizability, communicability, and memorability is enhanced.^11^

The societal benefits take the form of family and group cohesion, transmission of the group’s culture, sexual bonding, food preferences, and collective security. Tastes and odors can signal food quality; the mother’s voice can have a soothing effect on a child; erotic effects of images and odors can stimulate procreation; and the rousing effects of martial music can promote solidarity and common purpose.

These benefits may explain the evolutionary origins of the emotion-tagging powers of odors, melodies, and so forth. Levitin (2006) explained in more detail how biologically significant memes and their emotional valences can benefit the group.

## Priming and Potentiating Factors

### The Pervasiveness of Priming in Biology

Most biological systems, ranging from individual neurons to societies and nations, respond to a synergetic stimulus complex only when they have received some preparatory priming. When a neuron’s state of excitation has reached a certain threshold, it is primed to fire in response to a triggering event; sexual orgasms occur only when a state of arousal has been primed; and nations go to war only when tensions have been primed.

Aesthetic responses, similarly, require priming. For synergetic interactions to occur, the synergetic stimulus must be in place and the audience’s state of readiness must have been primed. In general, the biological utility of priming is that it enables rapid responses when speed is advantageous. It reduces the time and energy that the real-time response will require by using off-peak time for preparation. The resulting savings can make the difference between effectiveness or ineffectiveness of the stimulus and thus the occurrence or nonoccurrence of the response.

### Forms of Priming

#### Priming Based on Physiology and Widely Shared Life Experience

Many aesthetic effects in the arts rely on the neurally hard-wired noncognitive perceptual elements of the visual and auditory systems—priming via genetics. The human visual system decodes visual stimuli through multiple stages of processing, proceeding from the retina to the lateral geniculate nucleus to a succession of relays in the primary visual cortex and beyond, where such perceptual elements as lines, edges, corners, contours, borders, orientation, color, motion, brightness, contrast, outline, movement, flicker, shape, spatial positioning, and duration are identified (e.g., Kandel, 2012, Chapters 14 and 15).

Similarly, the physics of sound and the physiology of the auditory system inform human responses to rhythm, harmony, pitch, timbre, loudness, and direction of the sound (Levitin, 2006). These are the most universal conceptual elements that configure our perceptual responses in vision and audition, and function as the physiologically hard-wired perceptual elements that enable us to respond adaptively to the environment.

Also widely shared, though probably mostly learned rather than hard wired,^12^ are our myriad pan-cultural concepts: those that involve the earth’s natural environment—gravity, wetness, hot and cold, fire, wind, faces, voices, pain and pleasure, life and death, hunger and thirst, and the many temporal, spatial, causal, and logical relations (and/or, if/then/unless, therefore, but not, etc.). These and many other near-universal concepts are primed by the ordinary life experiences of most of the world’s population.^13^

Less widely shared types of priming are culture-specific learning experiences. For instance, only some audiences would be primed with the culture-related concepts in Picasso’s bicycle bull sculpture—the bicycle parts, the features of a bull’s head, and the fact that bulls are largely unrelated to bicycles. The synergetic brews that give rise to aesthetic responses feature both the physiologically hard-wired perceptual responses and the culture-specific responses.

#### Priming Via Exposure to a Work

One reason great works of art are not aesthetically accessible to everyone is that not everyone has had the priming histories with respect to the required concepts and their relations. Full appreciation of great works of art requires a certain amount and type of exposure to the work itself, or to similar works (Marr, 2003). Most great pieces of music must be heard multiple times; great paintings require viewing time; and great poems must be studied to be appreciated fully. It is during such exposure that the necessary concepts and relations can attain the functionality thresholds required for the intended synergetic interactions and aesthetic impact.^14^ Additional exposure to a work can augment its aesthetic effect by recruiting additional elements into the brew of synergizing elements.

### The Priming of Paths Through the Concept Repertoire

When Archimedes jumped out of his bathtub and ran down the street shouting, “Eureka! Eureka!”, he had presumably connected two distantly related concepts: the rise in his tub’s water level when he got in, and a method for determining the purity of the gold in the king’s crown—the amount of silver that may have been mixed in with the gold. He may also have realized that he had discovered a principle that applies to all problems of that type (Fields & Moss, 2008).

This story implies that Archimedes’ entry into the tub raised not only the water level but also the priming level of one or more concepts whose strength was not yet sufficient to light up the entire connecting path. A final bit of priming raised that level, thereby short-circuiting the entire 12-link chain. Here is a possible chain of concepts and relations in Archimedes’ concept repertoire that might have been short-circuited:

1. The water level in the tub rose when he got in.
2. The amount by which water level rises multiplied by the surface area equals the volume of water displaced.
3. The result is the volume of the submerged part of his body—an irregularly shaped object.
4. The amount of water displaced is a measure of the volume of *any* irregularly shaped object.
5. The crown is an irregularly shaped object.
6. The amount of water the crown displaces is equal to its volume *V*.
7. Weight divided by volume equals density.
8. The crown’s weight is *W*.
9. So, the density of the crown is *W/ V*.
10. The known density of pure gold is *G*
11. The known density of silver is lower than *G*
12. Comparing *W/V* to *G* reveals the amount of silver, if any, that may have been mixed in with the crown’s gold.

#### Leaps Through the Concept Space

The same path-illumination process can explain how an audience can make connections between distantly related concepts. In murder mysteries, the author primes a multiple-link connection by relating the story in a way that primes the surprise effect of the final denouement*—*the “Aha!” that the murderer was the person the reader least suspected.

But the chain is rarely as long as the one that prompted Archimedes’ epiphany. It can be as short as a single link (the limiting case of a chain), as in Picasso’s bicycle bull.

In general, four factors determine how readily a potential connection between distantly related concepts will form:

- The length of the chain of relations that connects them. 15
- The strength of the concepts that make up the chain (Arntzen et al., 2015).
- The level of priming of the chain’s links.
- The reinforcement value (or utility) of the potential connection.

### Factors that potentiate synergetic interactions

As explained in Part 1, aesthetic responses require certain potentiating factors for their occurrence. Among these are the potentiating actions, the behavioral state of the audience, sociocultural factors, and a trigger for the response. Beyond being primed with respect to the ingredients of the synergetic brew, the audience must also be engaged—not otherwise preoccupied by distractions or incompatible emotions—and sufficiently attentive, receptive, and aroused.

Once potentiated, some aesthetic reactions are virtually instantaneous, like those to faces, certain sounds, or certain tastes. Others, like responses to the synergetic brews of poems, literature, paintings, or humor, have much longer latencies, and some works require multiple exposures before an aesthetic response is evoked. The reason for these differences resides in the time it takes the audience to respond to the elements of the stimulus. Elements that involve cognitive processes like recollection, recognition, or other types of processing have longer latencies. Some elements must actually be learned before they can function as part of the synergetic brew.

Though aesthetic responses are essentially emotional and covert, they are always potentiated by operant behavior that sets the occasion for their occurrence (Section 1.4). Sociocultural factors, too, normally contribute to the behavioral contingencies that potentiate aesthetic responses. For instance, an aesthetic response is more likely to occur in the presence of other individuals from the same culture who share the relevant priming history or would support a certain type of response. As previously indicated, factors that potentiate aesthetic reactions act somewhat like the catalysts that potentiate chemical reactions.

### Culture as a Potentiating Factor

In human societies, the arts function as a medium for sharing perceptions, information, and beliefs. Even the earliest human art forms (ornamentation, ritual, dance, graphics, symbols, flags, music) are likely to have served as media for defining the cultural identity and promoting the internal cohesion of the social group (e.g., tribe, clan, village). Shared aesthetic responses play a prominent role in the self-definition and cohesive glue of human societies in general—families, communities, organizations, and entire nations (Dissanayake, 2008).

In the context of cultures, aesthetic phenomena, like a language, may be viewed as “memes”—the cultural counterpart of genes (Dawkins, 1989; Glenn, 2003). The term refers to behavior patterns or concepts that are widely shared among the members of a group or culture. Examples of aesthetic memes in Western cultures are French postimpressionist art, 18th-century central European classical music, and 20th-century rock and roll music. Individual members of each of the associated cultures would have a greater tendency than members of other cultures to respond aesthetically for each of these memes.

Memes contribute to the formation of metacontingencies (Glenn et al., 2016) and metacontingencies in turn contribute to the formation of memes (Glenn, 1991). Networks of aesthetic memes create the metacontingencies that promote internal cohesion of societies and cultures, thereby perpetuating the cultural host group’s embedded aesthetic memes. Monod (1971) pointed out that memes, like genes, share many properties of organisms. Evolutionary mechanisms perpetuate both genes and memes, in both cases via perpetuation of their hosts—individuals and social groups, respectively.

### The Diversity of Aesthetics Memes

The metacontingencies to which memes contribute thus participate in potentiating and priming the aesthetic responses of the individual members of the group. Consideration of distant or alien cultures reveals the enormous diversity of art forms and their memes, and hence of the diverse aesthetic propensities of these cultures’ members.

Examples of such alien-to-us art forms are the ancient Aztec culture’s cannibalistic rituals and cuisines, and the head-shrinking arts of certain ancient tribes of New Guinea and South America. Foods that are considered delicacies in one culture may be considered revolting in another. Similar culture-based divergences of aesthetic tastes pervade music, the visual arts, dance, literature, and other disciplines. Rare is the aesthetic taste that is untainted by the priming effects of sociocultural factors. In fact, the influence of such factors can be so great as to overwhelm the contributions of the stimulus itself (viz. *The Emperor’s New Clothes*). The less an individual is primed in the elements of the pertinent art form or discipline, the greater the relative contribution of the sociocultural factors.

## Devices Used in the Creation of Aesthetic Effects

### Repetition and Its Biological Significance

When a grazing deer hears an unfamiliar sound, it may raise its head and perk up its ears. If it hears it a second time, it bounds away. When a scientist proclaims a significant discovery, others seek its replication. Repetition is generally confirmatory. One wants to know, “Is it real?” “Is it true?” “Does it warrant action?”

Repetition is a widely used attention-directing device. Saying “I repeat…” calls attention to the repeated element. When we knock on a door, we usually knock more than once; many pieces of classical music begin with two or three repetitions of a musical figure; and the designs of decorations often use repeating elements.

Yet the effects of repetition are not limited to attention getting or confirmation. They also create expectation. When the repeating stimulus signals a certain learned consequence, whether positive or negative, the effect of further repetitions, beyond two, depends on whether the expected next event occurs. The deer may not wait for a third repetition to find out. But in human situations where further repetitions do occur without the expected outcome materializing, the initial effect is surprise, creating a potential for aesthetic impact. If repetitions continue beyond that point, this potential diminishes, and the eventual result is habituation or extinction of the stimulus' effect.

### Symmetry

Symmetry is generally associated with mirror images, but the formal definition is that a system is said to have symmetry when its attributes are conserved in a transformation (Marr, 2013; Petitjean, 2007; Weil, 1952). But even when attributes of a stimulus are conserved in the physical or mathematical sense, they are not necessarily conserved in the behavioral sense: The eyes cannot focus on both sides of a mirror image simultaneously, and one cannot take both forks in a road—these are behavioral forms of “symmetry breaking.”

Symmetry breaking may direct an audience’s attention to the altered or conserved elements, which can then serve as ingredients of synergetic brews. The audience generalizes or discriminates among corresponding attributes according to their alteration or conservation in the transformation.

The simplest visual transformations are reflections, inversions, rotations, and scale changes. The more complex and elaborate the transformation, the greater the opportunity for surprise when the viewer identifies the constancies (kaleidoscopic images are a rudimentary example), and the greater the potential for contribution to a synergetic brew.

### The Use of Symmetry in Synergetic Brews

A stock artistic device is to create minor deviations from the regularities and predictabilities of symmetry. The resulting disconfirmations of expectations can contribute to the surprise effects.

Examples of symmetrical transformations in the arts: The meter and melody of one stanza of a song or poem, or the rhythm or tonality of one measure of a piece of music, is conserved in the next stanza or measure; in the A-B-A musical form, slight variations in the second *A* invite comparison with the first; in visual art, regularities and repetitive patterns of color, texture, and shape create expectations as the eye moves across a painting; dance performances create expectations via repeating body movements. Ceramics, weaving, and beading, all use repetition and symmetry effects. All of these examples illustrate how symmetrical transformations can set the occasion for discrimination and generalization, with resulting opportunities for non-confirmation of expectations and surprise via the manipulation of concepts. The resulting learning effects can be reinforcing.

Another reason symmetry is a common ingredient of synergetic brews in the creation of aesthetic effects is that symmetry tends to be associated with desirable attributes: in animals, with fitness and health; in abstract domains, with correctness, as when two sides of an equation balance; in logic, with equivalence; in psychology, with mutuality, reciprocity, dialog, and tit for tat; and in architectural structures, with stability. If the Taj Mahal were shown as left side only or the Eiffel Tower as one of its quarters only, with symmetry thus removed but all geometric information conserved, their aesthetic impact would be questionable.

### Descriptions of the Devices

As stated in Section 1.10, a limited number of devices appear to account for all of the more than 200 observed effects described in Parts 8 and 9. Every art form and discipline, and every effect, uses a different combination of the 16 devices described below to create synergetic brews whose elements are arranged so as to interact to generate aesthetic responses for appropriately primed individuals. These devices, which can be likened to a chef’s recipes, always involve manipulation of concepts and their relations in the audiences’ concept repertoires (Part 2).

- {a} *Combining concepts* to create surprising new concepts and form new relations among them, or to expand the classes that define them. This device, one of most widely used, can involve placing a concept in a new context; arranging its temporal or sequential placement, as in music or literature; or its spatial placement as in visual arts.
- {b} *Nonrealization of an expectation—*This device, also one of the most widely used, involves the priming of an expectation of a certain continuation, outcome, or relationship (or taking advantage of a learning history that does) and then presenting a different continuation, outcome, or relationship, thereby creating surprise.
- {c} *Reclassification—*Identifying non-obvious and unrecognized commonalities among existing concepts (Bush, Sidman, & de Rose, 1989; DeRosse & Fields, 2010), as in recategorizing whales as mammals when they had been categorized as fish—another form of concept formation.
- {d} *Distortion or exaggeration of an attribute—*Devices that evoke discrimination of, or that focus attention on, certain concept features or conceptual discrepancies, thereby making these available for manipulation.
- {e} *Distillation or summarization*—Identification of essential features and elimination of nonessential ones. Where used: caricatures, mathematical theorems, scientific theories, literature, musical compositions (as in *codas*); or simplifying assumptions in science.
- {f} *Provoking induction*—Inducing generalization and expansion of a class that defines a concept; and identifying additional instances of the concept, or additional applications of a relation among concepts. Where used: parables; similes; poetry; scientific theories and mathematical theorems; pantomime and mime performance.
- {g} *Linking distantly related concepts* by priming the required connecting links within the audience’s concept repertoire (Section 6.3); creating linkages between concepts that may be remote from each other within the concept repertoire.
- {h} *Recognition—*Evoking a concept by presenting a subset of the class that defines it, or stimuli that evoke its recollection or associations, as in concept refreshment. Where used: caricatures; musical forms like fugues, theme and variations, and leitmotifs; literary or poetic allusions; dance and other performance arts; film.
- {i} *Mirroring of emotions—*Displays of emotion by the character of a film, story, play, or opera, or by an actor, dancer, orator, or musical performer. When the audience mirrors emotional displays, an emotional element is added to the synergetic brew (Part 5).
- {j} *Emotionalizing concepts—*Stimuli that are emotionalizing by virtue of their biological or cultural significance. Examples include frightening concepts, depictions of violence, certain facial expressions, religious themes, sexual connotations, hostile or hurtful interpersonal interactions, voice effects, hugeness, very loud sounds, dissonances. Where used: in film, visual arts, music, dance, poetry, literature, and architecture.
- {k} *Repetition* of a concept or stimulus can draw attention to it, increase its prominence, or confirm the preceding concept (Section 7.1); repetition can create expectations whose nonrealization can occasion surprise (Section 4.2).
- {l} *Symmetry* (Sections 7.2 and 7.3) can focus attention on features of concepts that are conserved in the face of transformations, whether musical, visual, literary, or conceptual, and thereby create expectations whose non-realization can occasion surprise (Sections 4.2 and 4.3). Symmetry also tends to be associated with desirable attributes like fitness, physical stability, or correctness of equations.
- {m} *Parsimony—*Achieving a result or effect with a surprising economy of means, effort, or other resources (Section 4.7). Where used: caricature, dance, music, laws of physics, mathematical theorems, beautiful chess moves, literature.
- {n} *Humor* (Section 9.5)—Its many genres include jokes, irony, wit, comedy, parody, sarcasm, whimsy, slapstick, clowning, mockery, and puns. All rely on juxtaposition of concepts to reveal incongruity or contradiction. Humor’s reinforcing functions depend on coordination with other devices to displace emotions that are aversive (fear, anger, sadness, etc.), often by revealing hidden relations or other forms of class expansion.
- {o} *Audience involvement—*When the audience actively participates, elements are added to the synergetic brew (Section 4.10), thereby increasing the number of potential conceptual relations multiplicatively. Audience involvement can be particularly impactful in the performance arts, music, poetry, literature, visual art, architecture, science, mathematics, and games.
- {p} *Synergistic augmentation—*When any of the above devices are at play simultaneously, their combined effect can be greater than merely additive, that is, synergistic. Section 1.14 explained how the result of a synergistic interaction can interact synergetically with another element of a brew. Thus, the creation of synergistic interactions among the above devices is itself a device.

## Aesthetic Effects in the Arts

### Evidence for the Theory

The purpose of this Part 8 is to demonstrate the theory’s usefulness in analyzing and explaining aesthetic phenomena in poetry, literature, music, visual arts, performance arts, dance, culinary arts, architecture, and nature. The examples, chosen mainly for recognizability, illustrate the various ways the limited number of devices described in Section 7.4 are able to generate a seemingly unlimited range of synergetic brews and aesthetic effects. For every example, the *main* device or devices are identified by the letter(s) they are given in Section 7.4. The word *main* makes the point that other devices from the list are usually also at play, though their role in creating the observed effect may be less significant. Only the main devices are referenced for each of the aesthetic effects discussed. Note also that the “aesthetic” responses analyzed may be so subtle as to escape notice, like other subaesthetic reactions.

Readers may choose to scan Parts 8 and 9 just enough to convince themselves of the general applicability of the theory, focusing only on the sections that interest them particularly.

### Use of the devices in Poetry

Poetry provides some relatively pure examples of the creation of aesthetic effects created by use of the devices described in Section 7.4. Rhyme creates phonological relations and symmetries; meter creates rhythmic and structural ones. These can then interact with other concepts and relations. [Devices {a}{b}{k}{l}{p}]. Common synergetic effects of brews that contain rhyme- or meter-enhanced words are: an increase in the word’s prominence and emphasis. Structural and rhythmic regularities generated by rhyme and meter also increase the word flow’s predictability, thereby making it easier to learn and memorize.^16^ They also impart an impression of intentionality and appropriateness to the choice of the word, and of truthfulness to the statement in which the word is used. For instance, aphorisms that rhyme are more credible than the same ones with rhyme eliminated (McGlone & Tofighbakhsh, 2000). For the same reasons, rhyme and alliteration are used extensively in advertising.

#### The Basis of These Effects

Rhyme and meter have these effects because they involve repetition and the formation of new relations {a}{b}{k}{p}. Repetition is confirmatory (Section 7.2) and a concept is strengthened when its relations are expanded (Section 2.4). Rhyme and meter also create a flow of micro-expectations that the poet can violate to create mini-surprises {b}. Free verse poetry that lacks rhyme and meter relies on other, often more abstract, ingredients for its synergetic brews.

Music can add yet another dimension to a poem’s meanings (viz., nursery rhymes), as can prosody when the words are recited. Additional devices are analogies, similes, and metaphors, all of which work by class expansion of concepts, often in synergetic interaction with phonological, rhythmic, or musical stimuli {a}{f}{p}. A unique attribute of poetry is its relation to speech and performance art, as in poetry recitation and rap. The ability to speak in rhyme or rhythmic cadence projects articulateness and verbal fluency (Dutton, 2003). It has thus found a role in courtship, as when Cyrano impresses Roxanne by improvising rhymes while fencing—an illustration of how artistic and verbal prowess may have evolved from its primordial functions {o}.

#### Priming Surprises

In music, literature, poetry, and film, a common priming device is the presentation of a series of specific instances of a concept prior to its summary statement. The instances prime the audience to expect one type of summary or conclusion, while the one that actually follows is not the expected one {b}.

For example, in his poem *The Second Coming*, Yeats implies that things are bad: “The falcon cannot hear the falconer; /Things fall apart; the center cannot hold; /Mere anarchy is loosed upon the world” {l}. But the conclusion that the audience is now primed to expect is not the monstrous one with which Yeats surprises the audience {b}.

Another example is found in the opening scene of Goethe’s *Faust.* Faust enumerates the disciplines he has mastered (philosophy, theology, jurisprudence, and medicine). The reader might now expect Faust’s summary statement to refer to his erudition, but gets a surprise when Faust continues, “Here I now stand, poor fool, and am no wiser than before.” Some further instances of Faust’s misery prime the final surprise—the appearance of Mephisto {b}{l}{p}.

### Use of the Devices in Literature

#### Surprises That Span an Entire Work

In *Crime and Punishment*, Dostoyevsky develops the two facets of Raskolnikov’s personality through a series of incidents that involve him and Svidrigailov, his antithesis. By this concept formation method—contrasting instances and noninstances—Dostoyevsky primes the surprise of Raskolikov’s eventual transformation {b}{f}{p}. Murder mysteries and thriller movies provide other instances of the “final surprise” device {b}.

Aristotle, in his *Poetics* on tragedy, discusses how tragedians prime the final surprise, or *Katharsis.* The emotional impact, he explains relies on events that prime the audience’s emotions of pity and fear, events the audience must find realistic and credible, that is, they must mesh with its existing concept repertoires. This process primes the synergetic interaction and climax—for instance, that the man Oedipus had slain was his father and that the woman he had been sleeping with was his mother {b}{f}{j}{p}.

#### The Parable Device

A parable provokes generalization of its theme or message beyond the work’s specific characters and settings {f}{h}{p}. The effect is often gradual—a growing realization of the message’s generality due to the cumulative effect of successive instances of the concept that is being formed.

Metaphorical devices can help overcome readers’ resistance to transferring the message to themselves or their emotional discomfort, as when the message touches on moral or ethical topics. To bypass such resistance or discomfort, authors sometimes use characters and events that are safely distant from the reader’s personal experience. The fables of Aesop and La Fontaine feature animal characters that talk and interact in human ways, as does the wolf in *Little Red Riding Hood* or *Three Little Pigs*. If the characters were ordinary people, behaving in ways that strike too close to home, the reader might take the intended message too personally too quickly, and reject it.

But the use of the talking animal device is not just a Trojan horse against self-reference. It also invites generalization and concept formation, with a progressive but sufficiently gradual transfer from animal to human as events unfold. The synergetic interaction occurs at the moment of generalization, when the render realizes the generality of the characters’ behavior, {f}{h}{p}.

#### Other Generalization-Provoking Devices in Literature

Shakespeare used the same devices {b}{f}{h} in most of his plays. Rather than talking animals, he used kings, princes, royalty, or a *Moor of Venice*—characters who, like talking animals, are safely distant from the lives of ordinary theatergoers. Ancient Greek theater achieved a similar result by using masks, as if to say, “This character could be anybody.” When watching Oedipus Rex (note the king device again), the main synergetic impact occurs at the moment of generalization {f} {h}{l}{p}.

Some classical novels, too, are parables that provoke generalization through settings and characters whose metaphorical roles often become evident only gradually. Well-known ones from the classics are *Gulliver’s Travels, Don Quixote, Moby Dick, Animal Farm*, and *Lord of the Flies.* The same devices {d}{f}{h}{l}{p} are used in science fiction, space fantasy films like *Star Trek*, *Star Wars*, and other fantasy media that seek to convey messages via induction {f}. Their unrealistic, unnatural, and/or artificial settings provoke generalization and conceptualization.

Folk sayings and aphorisms use the same induction-provoking principle {f} but in a different context. “A stitch in time saves nine” obviously does not apply only to seamstresses, and “The early bird catches the worm,” or “Birds of a feather flock together” are clearly meant to apply beyond ornithology. The persuasiveness and aesthetic effects of aphorisms are also enhanced by the synergetic truth-imbuing effects of rhyme discussed earlier {f}{h}{p}.

### Use of the Devices in Music

The examples cited below illustrate how the devices described in Section 7.4 create synergetic brews and aesthetic effects in music. Though the examples are likely to have more associations for musicians, they may also be accessible to nonmusicians who are familiar with music.^17^

#### Beautiful Melodies and Harmonic Progressions

In listening to music, aesthetic responses generally occur at points where something unexpected and surprising happens, like a modulation (transition from one key to another) {b}. The composer may create synergetic brews by combining the modulation with a simultaneous unanticipated rhythmic twist, change of instrumentation, or other deviation from the listener’s expectations regarding melody, harmony, or timbre {a}{b}{p}. For example, in the third movement of his *Great C Major Symphony,* Franz Schubert primes a surprise by repeating the C major key’s root note C, alone and without harmony, several times in a row. Since any note played alone can serve as a bridge to any one of many other keys, the key actually chosen comes as a surprise {b}. The final C is the bridge to the new key of A flat major, thereby revealing the C’s double meaning. The simultaneous change to horns and slowing of the tempo is spine-tingling {a}{b}{p}. Beethoven employs this same modulation in the *Choral* movement of his *9th Symphony*, where the final A major chord provides the major third of a rousing F major chord, thereby modulating into F major for the march that follows. Verdi used this same modulation to introduce the *Anvil Chorus* in *Il Trovatore.* In all three of these examples, the synergetic interaction effect is created by combining the surprise of the modulation with a simultaneous change of instrumentation, timber, and rhythm {a}{b}{p}.

The evolution of European music over the centuries, from medieval liturgical music to today’s genres, has seen an increasing complexity of harmonic progressions, modulations, scales, and rhythms. It is possible that this evolution is due to composers’ need for new musical effects that can still achieve surprise in the face of the increasing familiarity of traditional ones. The aesthetic impact that a particular genre achieves depends entirely on the audience’s state of priming (Section 6.2).

#### Surprise Effects Based on Rhythm

Why is rhythm the indispensable ingredient of so many synergetic brews? Because it can prime short-term expectations and surprises through syncopation, acceleration, deceleration, pattern repetition, and so forth. The temporal patterns of rhythm provide an infinite range of opportunities for the violation of expectations via systematic deviations from regularity {b}{k}{l}.

Rhythm also identifies and demarcates musical units. The regularities of a rhythmic scaffolding can create expectations that the composer can then manipulate and violate for the achievement of surprise {b}{k}{l}{p}. Synergetic brews that use rhythm as an important ingredient include Stravinsky’s *Rites of Spring* and the syncopations of much Latin dance music.

#### Theme and Variations

In the classical genres, the theme on which the variations are based is stated at the outset. The variations share some combination of the theme’s identifying features—its harmonies, rhythms, and melodic elements, and present them for recognition in new contexts {a}{d}{h}{b}{p}.

#### The Canon and the Fugue

The canon is a form where two or three identical renditions of a musical passage play simultaneously but with staggered starting times. When different segments then overlap, surprising new harmonies are created {a}{k}{b}{p}. A similar device is used in fugues, but there, the original theme keeps changing. It is expanded, inverted, transposed, modulated, and embedded in surprising new contexts. The listener’s “Aha!” Is triggered by recognition of the theme in its altered forms, interacting synergetically with other elements {a}{d}{h}{p}.

#### Repetition With Context Variation

Much of traditional African music is based on refrains in which a basic musical concept is repeated in ever-varying contexts—rhythmic, vocal (call and response), and/or accompanying dance routines. When a musical unit keeps repeating in ever-changing contexts, its class expands {a}{h}{k}{l}{p}.

The *basso ostinato* in classical music achieves its effect by the same devices. A given bass figure is heard in ever-varying contexts, in each of which the repeating unit takes on different and sometimes surprising meanings {a}{l}. Same for the repeating chorus of a chorale. New contexts can be musical or textual, as in the stanzas and refrains of songs. Each new meaning thus generated serves as an additional ingredient of a synergetic brew.

#### The Development Section

In the classical sonata form, the development section combines identifying features of the first and second themes to synthesize new musical concepts. The listener experiences mini-surprises on hearing and recognizing the themes’ features in their altered forms, combinations, and contexts {a}{d}{h}.

A familiar example occurs in the first movement of Beethoven’s *5th Symphony*, where the first theme, “*ta, ta, ta, taah*,” (twice repeated) {k} {l} reappears in inverted form as the bass figure of the second theme. The relations between the two themes are then developed throughout the first movement and then more in the third, always in surprising ways {a}{d}{h}{p}.

#### The Coda

Theme-and-variations, fugues, and development sections all use devices {a} class expansion, but codas often do the opposite: they summarize the musical concepts heard earlier. The impact is based on how the essence of the musical concepts is distilled and exaggerated with nonessential features omitted, as in a caricature {d}{e}{f}{p}. For example, in the coda of the first movement of his Piano Concerto No. 3 in C minor, Beethoven offers three levels of summarization, each one terser than the previous one, and the final one just five notes long; in the final measures of the first movement of his *9th Symphony*, he summarizes the movement’s main themes with a terse, eight-note synopsis {e}.

#### The Leitmotif

A leitmotif is a musical theme associated with a person, object, action, or concept like fate, love, or death. In operatic works, it is often used as a musical narrative, essentially a priming device that signals relations between concepts—a commentary on the meaning of events as they occur—what a character may be fearing, recalling, planning, or anticipating. An example of the synergetic effect this device can achieve is the unexpected joining of the “fate” and “contract” themes in Richard Wagner’s “Twilight of the Gods”^18^ {a}{g}{j}{p}.

### Use of the Devices in the Visual Arts

The concepts that generate effects in the graphic visual arts fall into two broad categories: (A) purely visual concepts, based on colors and color schemes, compositions, shapes, forms, patterns, repetition, and symmetry, whose aesthetic impact is largely independent of things that may be depicted (as in abstract art), and (B) concepts based on depictions of the world and the audience’s knowledge of it. The impressionist and post-impressionist genres of painting often achieve synergetic interactions of type (A) and (B) effects in the same work {a}. Portraits and landscapes may use wonderful color schemes (type (A)), and at the same time play on viewer’s history with respect to people and nature (type (B)) {b}.

For both categories (A) and (B), the main visual arts employ most of the devices described in Section 7.4. Monet, Renoir, Cezanne, and Van Gogh used device {a} when they combined type (A) effects that play on concepts involving color relationships, contrast and texture, the rainbow spectrum, and devices designed to move the eye in systematic ways, with the use of type (B) effects.

Example: The aesthetic impact of the swirls in many of Van Gogh's paintings are due in part to the contexts in which they are placed {a}—cypress trees, a starry sky, or rolling hills—things that are static in the real world. Since swirls imply movement, their presence in static structures is surprising, incongruous, and emotionalizing—a synergetic interaction effect {a}{b}{d}{h}{j}{k}{p}.
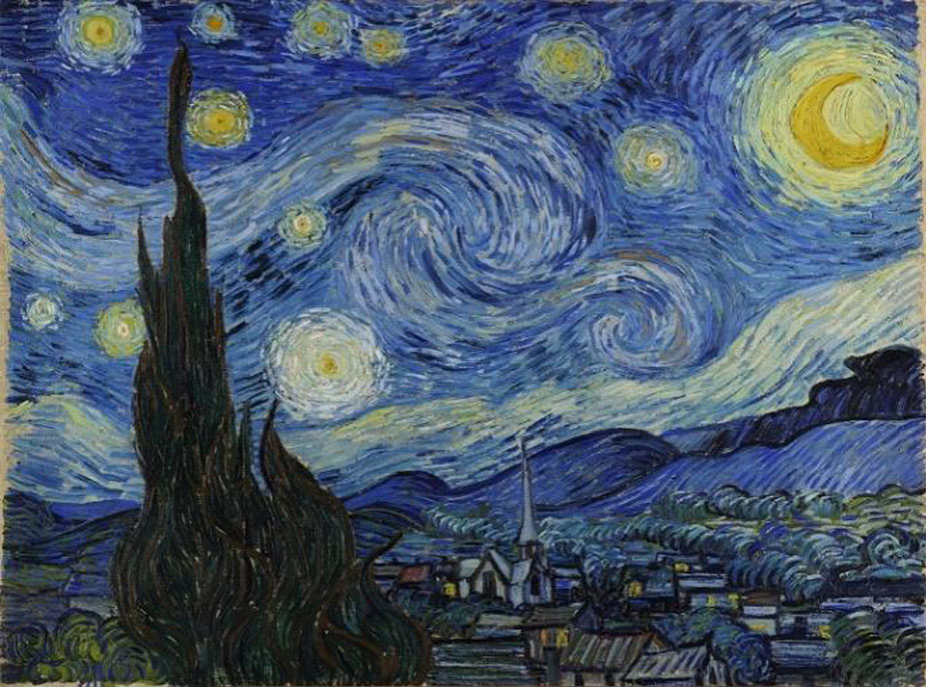


#### Rhythm and Repetitive Patterns

A device much used in visual arts is to repeat a shape, pattern, or color scheme while systematically varying a featured dimension, thereby focusing attention on that dimension and then expanding its class {d}{k}. Cezanne often used this device by means of side-by-side parallel brush strokes, each having a slightly different shape, texture, and color, in a progression that draws attention to the changing dimension. Calling attention to a dimension by juxtaposing exemplars that vary in that dimension progressively is analogous to the ways repetitive and rhythmic effects can direct attention in music and poetry {h}{k}{p}.

#### Type (B) Effects: Visual Commentary About the World

This category of effect relies on viewers’ priming histories: their knowledge of the world—what things look like, societal phenomena, beliefs, things that happen, and emotional connotations of these {b}{h}{j}{p}. Familiar examples of art genres that rely on such priming are portraiture, religious art, still lifes, political and social commentary, realism, surrealism, and caricature. Dutton (2003) explains how evolution, culture, habitat, and other types of priming can determine the biases and susceptibilities of audiences {b}{h}{j}{p}.

#### Relationship to Other Analyses of Visual Aesthetics

Most traditional analyses of aesthetics have focused on the stimulus and its perception. Oshin Vartanian, in discussing the contributions of Gestalt theory to the study of aesthetics, cites Rudolf Arnheim’s description of the stimulus: the symbolism in the placement of the figures in the *Isenheim Altar* painting—the colors of their garbs, their personal attributes, and the subtle symbolic interrelationships and meanings of these (Arnheim, 1974, p. 74; Vartanian, 2014). Elements of the synergetic brews are the succession of connections viewers may make as they notice or are informed of the various symbolisms. The synergetic interactions and their emotional effects occur if and when the viewer becomes aware of the interrelations among the symbols {a}{c}{g}{p}.

Eric Kandel (2012) examines the aesthetic impact of the artworks of Klimt, Schiele, and Kokoschka in the context of the Viennese cultural memes of that era—including medicine and Freudian psychology, these being some of the interacting elements of the works’ synergetic brews {a}{c}{g}{p}.

Similarly, the accessibility of much of the art of ancient or remote cultures depends on the audience’s pertinent priming history. But when the elements of a work transcend cultures and epochs by utilizing more universal concepts, like natural phenomena, faces, the emotions that facial expressions may convey, or hard-wired elements of the visual alphabet, the work’s aesthetic impact expands correspondingly across cultures and epochs.

#### Caricature

Caricatures generally seek to depict a particular person or type. A successful caricature identifies, usually by means of exaggeration, distortion, and/or elimination of nonessential features, a subset of the distinctive features sufficient for recognition {d}{e}{f}{h}{m}{p}. Many of the devices used in caricature are also used in other art forms and media, in musical *codas* or literary abstractions, summary statements, or mathematics.

Al Hirschfeld’s caricatures are remarkable for the way they identify many conceptual classes simultaneously—the essence not only of subjects’ facial features but also of their entire body, personality, unique way of moving, and in some cases, even their interactions and relationships with others {a}{e}{h}{p}. The synergetic interaction among those elements brings the subject to life. Hirschfeld’s caricatures owe much of their aesthetic impact to the economy of means with which they distill the essential elements {e}{k}{m} and their use of rhythmic repetitions of simple lines and shapes—spirals, swirls, and patterns, for purely visual aesthetic effects of type (A) {k}{m}{p}.

The evolution of European art over the centuries—from medieval religious painting to the multiple genres of modern art—has seen a proliferation of media, genres, and their hybrids. As in music, this evolution appears to be driven in part by the search for new ways to achieve synergetic effects and surprise in the face of viewers’ increasing familiarity with past genres.
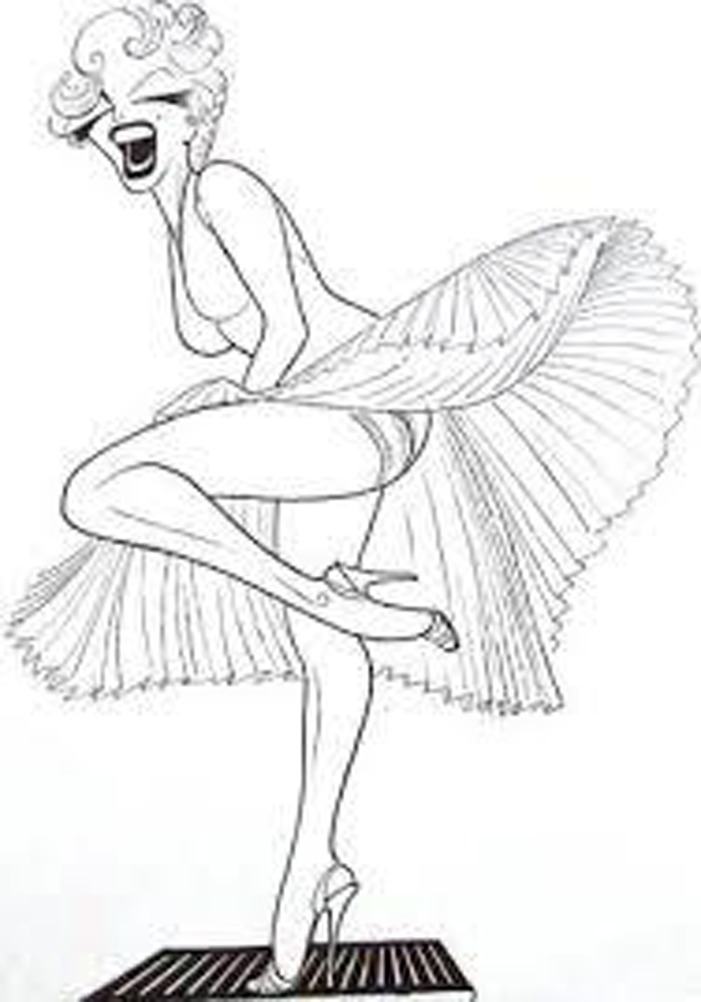


### Use of the Devices in Walt Disney’s Art

Many of Walt Disney’s creations are exemplars of synergetic brews that have emotional impact. Mickey Mouse’s main synergetically interacting elements are (1) neotenic features (large ratio of head-size-to-body and eye-size-to-face, high-pitched voice, asexual); (2) infantile clothing; (3) happy facial expression; (4) mischievous aplomb; and (5) good intelligence for a mouse, especially a young one {a}{d}{j}{p}. In Mickey Mouse movies, the synergetic brew is enhanced when motion, music, visual effects, and story lines, are added {p}.
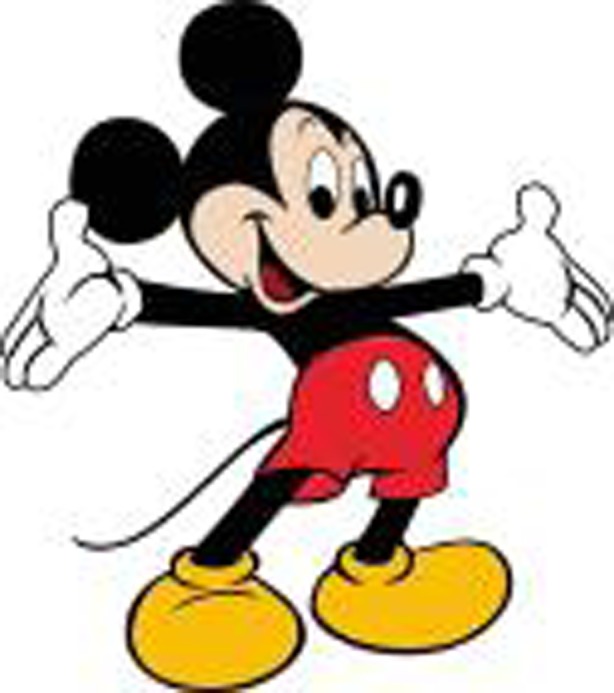


Mickey Mouse’s nonhuman embodiment increases its potential for the types of induction effects {f} discussed in Section 8.3. The emotional impact of the synergetic brew that is Mickey Mouse has permeated most of the world’s cultures during the century since its creation. But whether any given impact is an “aesthetic” one is a semantic question that will receive different answers from different audiences.

### Use of the Devices in Performance Art

Performers use emotionally expressive behavior to evoke emotional reactions from their audience, via mirroring (Part 5) {i}. The audience’s emotional response may then interact synergistically or synergetically with the music and other elements of the brew {a}{p}.

Orchestra conductors often seek to convey the music’s emotional meaning by expressive gesticulation, {i} as much for the audience’s as for the orchestra’s benefit. Rock and jazz performers may engage in vigorous and emotion-evoking antics; singers may act out the emotions they are trying to express; and instrumental performers use not just their fingers but also their heads, arms, and faces to convey emotion. In all of these cases, the audience responds to the performance emotionally in part through covert mirroring (see also Sections 5.3 and 5.4) {i}.

#### Dance

Dancers use their entire bodies to express nuanced emotions, and the audience’s response relies strongly on emotional mirroring {i}. Mime performances and their variants additionally rely on recognition and induction {f}{h}.

The dance medium is unique in its physicality—its use of movement as well as appearance, including attire and often decoration. The evolutionary roots of dance in primordial mating displays and performances are more evident for the dance medium than they are for any other art form. The medium’s erotic elements are often close to the surface or even the central theme.

Dance is also rife with synergistic augmentation among rhythmic effects, economy of effort (parsimony), and many of the other devices described in Section 7.4 {a}{b}{f}{h}{i}{j}{k}{m}{p}.

#### Magic Performance

Magicians use oral patter in combination with slights of hand and visual illusion to prime expectations that are then violated, thereby creating surprise {b}{c}{p}. Though the surprises thus generated are not validly instructive or informative from the reality standpoint, their pseudolearning effects are nonetheless reinforcing due to the surprise element.

### Use of the Devices in Architecture

In architecture, scale often interacts synergetically with design {a}. Table top models of the Eiffel tower, the vault of a cathedral, the Taj Mahal, or the Sydney opera house, would rarely elicit the gasps of wonder that the full-scale outdoor versions do. The conjunction of design elegance, scale, and surroundings interact synergistically and synergetically to create aesthetic impact {a}.

Other synergetically interacting elements sometimes used in architecture are gravity-defying devices like cantilevering or unexpected ways in which light enters indoor spaces {b}. Some architectural styles achieve synergetic effects by juxtaposing natural features like water, rocks, or vegetation with man-made structures to form the new concept of dwelling amid nature {a}{b}{c}. Surrounding structures, too, may contribute ingredients to a synergetic brew.

### Use of the Devices In Culinary Arts

Chefs can achieve aesthetic effects by creating synergetic brews of flavors, textures, and visual appearance {a}. New combinations need to be primed by the audience’s food history, and yet be unexpected and surprising, like crunchiness of a normally smooth food or sweetness in the case of meats {a}{b}{c}{p}. Flavors and textures can also contribute to the synergetic brew merely by refreshing memories, as in the way refreshment occurs in other arts (Sections 4.5 and 4.6). Odors and flavors also often have strong emotional associations {a}{j}.

### The Devices In Natural Aesthetic Phenomena

In the instances where the aesthetic brew is not man-made but natural, subsets of the same 16 devices are at play. I will continue to use the term *device* despite its connotation of a human agency.

#### *Sunsets—*Main elements

Type A effects, consisting of the brightly lit portion of sky, the juxtaposition of complementary colors due to the decomposition of the light spectrum by diffraction, rhythmic progression patterns due to atmospheric phenomena, the scale and three-dimensionality of the stimuli, suggestive patterns created by cloud formations, and color saturation gradients as a function of visual distance from the sun {a}{j}{p}{k}{l}.

#### *Flowers—*Main elements

Type A effects consisting of three-dimensional designs that feature symmetry, geometric rhythmic regularity, simplicity, repetition, highly saturated colors in the part of the color spectrum often opposite and complementary to the green of the surrounding vegetation, and fragrances whose reinforcing properties are largely nonlearned {a}{j}{k}{l}{m}{p}.

#### *Butterflies and bird plumages—*Main elements

Type A effects, including symmetry, elaborate color schemes, sophisticated designs, movement routines that create expectations, and in some butterfly species, Type B effects that evoke or mimic eyes of predators, bird feathers, or vegetation {a}{j}{k}{l}{m}{p}.

Other natural synergetic brews to which the term *beautiful* is commonly applied are landscapes, mammals, human bodies, crystals, marine life, and exoskeletons of marine animals.

## “Aesthetic” Effects in Other Disciplines

### Semantics Issues

The quotation marks around the term “aesthetic” are intended to convey that in the disciplines discussed below, the semantics of the terms *aesthetic* are stretched, as are those of the adjective *beautiful*. In Western cultures, the semantic ground zero for these terms have tended to be the visual arts, music, and poetry, with literature not far removed. Those arts are discussed in Part 8. As we draw away from this ground zero, new adjectival components join the profiles, and those are discussed in this Part 9. In the case of humor, for instance, the term *funny* becomes prominent. For horror movies and thrillers, *scary* is common. For mathematical theorems or scientific theories, such terms as *elegant* make their entry. Terms sometimes applied to architectural structures are *awesome* or *grandiose*; to culinary works, *delicious*; to human bodies, *graceful* or *sexy*; to actions of people, *admirable*.

But in addition to these familiar aesthetic domains of Western cultures, consider just those of Japan: In addition to a rich trove of musical and visual art genres, there is bonsai, many genres of martial arts, ceramics, calligraphy, landscaping, flower arrangement, the tea ceremony, costumes, architecture, Noh plays, Kabuki theater, and puppetry. To bring into perspective the vast range of the world’s arts and disciplines, multiply just those of Japan by the world’s thousands of other cultures, each with its own nested sets of aesthetic domains within domains.

Like Part 8, this Part 9 is intended to demonstrate the generality of the present conceptualization of aesthetics and the applicability of the same small set of devices to create all of the identified effects. The point is that the adjectives applied to the effects depend on the semantics of the prevailing culture, while the devices that create the synergetic brews for the effects remain the same.

Again, readers may choose to scan this Part 9 for items that interest them.

### The Devices in Film

Section 1.7 described the unique power of film to control an audience’s behavior, and Section 4.1 discussed film’s reinforcing power. This section analyzes the dependence of these powers on synergetic effects.

Here are some examples. The film medium can create synergetic brews that impart new meanings and emotional impact even to some of the greatest works of classical music. One instance is the way the second movement of Mozart’s *21st Piano Concerto* acquires new meanings as it interacts synergetically with the story in the movie *Elvira Madigan* {a}{h}{j}. Another is the 1941 Disney movie *Fantasia,* 19 where Goethe’s ballad *The Sorcerer’s Apprentice* interacts synergetically with visually impactful cartoon scenes, the music of Paul Dukas, and Mickey Mouse’s performance {a}{f}{h}{i}{j}{p}. Other stunning synergetic brews in *Fantasia* are amalgamations of musical masterpieces with visual art and emotionalizing dramas like “Night on Bald Mountain,” extinction of the dinosaurs, and scenes from Greek mythology, all achieved by devices {a}{b}{f}{i}{h}{j}{m}{p}.

### Impact as a Function of Number of Synergetic Ingredients

The history of film since 1900 demonstrates the effect of adding one synergetic ingredient at a time, one every few years, to the synergetic brews of film—a unique natural experiment: motion was introduced first; then sound; then drama and plot; then sophisticated acting; then color; then enhanced visual detail; then cinematographic technology; then screen size; then stereo sound; and then 3-D. The history of the film industry attests to the incrementally cumulative transformative, reinforcing, and emotionalizing impact of each of these additions.

The sheer number of distinct elements that comprise the brews of the film medium suggests that this large number may be responsible for the medium’s great control over its audiences. Motion pictures are created by teams that usually include screenplay writers, directors, actors, cinematographers, music directors, and many others. Video game development similarly requires the efforts of a team, with every team member contributing a vital element to the brew. The fact that these media require the efforts of large teams to create the brew attests to the unusually large number of interacting elements. In any given scene, as many as eight of the devices described in Section 7.4 may be at play simultaneously, the most commonly used ones being {a}{b}{c}{d}{f}{h}{i}{j}{k}{m}{p}. The brews of the film medium bring together more synergetically interacting elements than those of any other discipline.

This observation suggests the testable hypothesis that the magnitude of a synergetic brew’s impact is a function of the number of its interacting elements. But the completeness with which the film medium is able to capture and control an audience’s behavior and emotional involvement, to the point where it is rendered oblivious to its surroundings, suggests the possibility of a second, supertransformative effect—a cusp that may occur when the number of synergetic devices at play or elements in the brew reach a certain critical point. Again, fMRI technology may be a useful tool for exploring such a possible effect in terms of its neural correlates.

### The Devices in Video Games

Though usually not as rich in media resources as motion pictures, the active and interactive aspect of video game play adds the powerful reinforcing and instructional effects of learning via interaction with a responsive environment, immediate feedback, and control of consequences. Since video game play usually requires the development of a game-specific skill, the development of such skills, like all learning, is one of the player’s sources of reinforcement, regardless of the transferability of these skills to nongame settings. Another source of reinforcement is created by schedules that sprinkle small successes amid more numerous non-but-near successes {a}{b}{c}{d}{f}{h}{j}{k}{l}{m}{p}.

### The Devices in Humor

There are evidently many types of humor (see also Sections 3.5, 4.10, and 7.4 device {n}). The synergetic interaction termed humor occurs when concepts are juxtaposed, often with some preparatory priming, to create a surprising incongruity, discrepancy, or contradiction. Koestler (1964) quotes a joke that illustrates some of humor’s devices:

A Marquis at the court of Louis XIV who, on entering his wife’s boudoir and finding her in the arms of a Bishop, walked calmly to the window and went through the motions of blessing the people in the street. “What are you doing?” cried the anguished wife. “Monseigneur is performing my functions,” replied the Marquis, “so I am performing his.” (p. 96)

The audience is primed to seek an explanation for the Marquis’s calm demeanor—which is incongruous with the anger one might expect—as well as his incongruous blessing movements {b}. The answer arrives in the punchline, when the Marquis collapses four incongruities into 10 words: *Monseigneur is performing my functions so I am performing his.*

- He characterizes the Bishop’s adulterous activity as a “function,” thus juxtaposing it against the Bishop’s official religious functions {b}{c}{n};
- he implies that the Bishop’s religious functions are interchangeable with a husband’s sexual functions {b}{c}{n};
- he pretends to perform a religious function even though he is a Marquis, {b}{c}{n}, and
- he displays calm logic in contrast to the rage one might expect, an incongruity similar to the deadpan demeanor of comedians {b}{c}{n}.

In some types of humor, the punchline is created by the audience when it perceives the incongruity, discrepancy, or double meaning (“gets it”) {c}. When Joe says to Jane, “Let’s talk in rhyme today,” and she responds, “Oh no, I hate to talk that way!” the synergetic interaction occurs when the reader perceives the contradiction between her use of rhyme and the meaning of her answer. The whimsical humor of Picasso’s bicycle bull, too, resides in its double meaning, as in a pun, often augmented by parsimony {b}{c}{m}.

All of these examples illustrate the three devices present in most humor:

- Priming of certain audience expectations {b}.
- Juxtaposition of concepts that are incongruous or contradictory {c}.
- Presenting these simultaneously so that they may interact synergistically or synergetically {p}.

Some of these humor devices have also been described by others (e.g., Goldstein & McGhee, 1972).

### The Devices in Science

The aesthetic impact of simple equations, like *F* = *ma* (Force = mass × acceleration), *E* = *IR* (Voltage = Current × Resistance), or *E* = *mc* ^2^, is based on their power to describe and predict an infinite range of events that hold true throughout the universe—extreme instances of power amplification and parsimony {m} (see Sections 4.7 - 4.9). In all of these formulas, parsimony is a key ingredient of the synergetic brew.

The association between aesthetic impact and “truth” (in the scientific validity sense of the term, “true in the real world”) is reflected in the empirical observation that parsimony suggests truth, and truth tends to be parsimonious (Section 4.8).

### The Devices in Mathematics

The parsimony of the Pythagorean theorem, *a* ^*2*^ + *b* ^*2*^ = *c* ^*2*^, resides in the surprising economy of means by which it predicts the length of any right triangle’s hypotenuse based on the lengths of the triangle’s legs, in spite of the infinite range of their possible lengths. And there are, of course, untold numbers of far more sophisticated instances of aesthetically pleasing theorems.

In mathematics, surprise is often occasioned by linkages between concepts that may at first seem unrelated {a}{c}{g}. A simple example is the cumulative summation of consecutive odd integers 1, 3, 5, 7, and so forth. The first two add up to 4, the first 3 add up to 9, the first 4 to 16, the first 5 to 25, and so forth. How come they always add up to a perfect square {b}?

Even more surprising is the geometric explanation. Draw one dot. Then add 1 dot next to it and 2 dots under those 2 to make a 2 × 2 square. Then add 2 dots to one side of that square and 3 dots under it to make a 3 × 3 square. Then add 3 dots to one side of that one and 4 dots under it, to make a 4 × 4 square. To create those squares, the number of dots you add is always the sum of the next two consecutive numbers—1 and 2, 2 and 3, 3 and 4, 4 and 5, and so forth. Their sums (3, 5, 7, 9, etc.) are always the next odd number. That is why cumulating consecutive odd numbers preserves the perfect square {b}{f}.

This physical demonstration creates a surprising connection between seemingly disparate concepts—a numerical one—odd numbers adding up to perfect squares—and a *geometric* one that reveals the reason they do. The synergetic brew formed by these concepts may create a type of aesthetic impact that an appropriately primed audience may call elegant or parsimonious {c}{f}{g}{m}.

### The Devices In Chess and Go 20

The explanation of the quasi-addictive appeal of games like chess and *Go* must include the highly reinforcing immediacy and emotionalizing effects of these games’ battles of ideas, the continuously generated succession of synergetic interactions and move-upon-move surprises. But what are the attributes that cause certain moves to be called “beautiful”? One is the move’s achievement of a desired result by violating generally applicable strategic principles (Margulies, 1977), like sacrificing material rather than maximizing it {a} {m}. Another is the projection of surprising power with minimal material resources, as when a single move creates or parries multiple threats {m}{p}.

A famous example is a chess game that Adolf Anderssen played in 1851. Often called “the immortal game,” it features a long sequence of sacrificial moves, each the only one that averts loss, and each leaving the opponent with only one good reply. In the game’s final position, Anderssen has only two knights and a bishop left, each performing its minimal function in the delivery of checkmate, while his opponent still had *all* of his pieces—the maximization of two types of parsimony^21^—economy of material and of function, {m} (Schenk, 2006).

In 1846 Japan, 17-year-old Shusaku played an amazing move against the *Go* champion, Inoue Gennan Inseki. Shusaku placed the stone in the middle of the board, far away from all the ongoing battle sites. Yet the stone projected its influence onto all of them. Again, parsimony {m}. In *Go* lore, the move is referred to as the “ear-reddening move” for its observed effect on Gennan, but it was only the audience that experienced the move’s aesthetic effect.

### The Devices In Actions of Individuals and Groups

Any type of event can function as an element of a synergetic brew, including actions of individuals. The annals of history are replete with examples of how synergetically interacting actions of a few individuals had cusp-like transformative effects—scientific discoveries and inventions that changed the world, the formation of national governments, or the initiation of wars (Malott, 2015, 2016).

When a team effort produces a transformative event like a great discovery, technological advance, or work (as in architecture, opera production, film making, or technology) {a}{b}{m}{p}, only audiences that have the priming history required for access to that achievement are likely to experience an aesthetic impact. Such audiences may have aesthetic reactions to the moon landing, the internet, gene splicing, fMRI, the GPS, or integrated circuit technology. All of these and countless other achievements of science and technology were the result of synergetic interactions among efforts of multiple individuals.

#### Effects Generated by Artistic Performances

Performers like dancers, figure skaters, acrobats, and sports virtuosos often create surprise by violating expectations as to what is physically possible {b}. Mirroring physically astounding performances can put the audience in touch with the gap between the performer’s abilities and their own {j}, especially when the performance seems effortless {m}{p}.

#### Effects Generated by Interpersonal Acts

In the interpersonal domain, we may call a person’s act “beautiful” or “magnificent” when it is surprisingly selfless and yet costly or painful to its agent; or where the act is kind or forgiving when we might have expected it to be vengeful or retaliatory {a}{b}{c}{i}{p}. In such cases the elements that interact synergetically are concepts relating to social behavior rather than to sensory ones as in music or art.

These types of social synergetic interactions are often featured in literature, film, and opera. The audience may experience the emotional impact generated by violated expectations when mirroring the displayed emotions {b}{c}{i}{p}.

### It Does Not Matter What We Call It

The examples presented in this Part 9 are intended to help make the point that regardless of whether the response is termed *aesthetic* or something else, what matters is the behavioral structure of the phenomenon and the devices that create it. The descriptive or adjectival term used depends on the speaker’s idiosyncratic semantic history and culture. Aesthetics, by whatever name, must therefore be considered a distinctive pan-cultural behavioral phenomenon deserving of study by the methods of science.

## Future Avenues of Inquiry

One goal of the naturalistic examination of aesthetic phenomena in Parts 8 and 9 was to show how a limited set of devices suffices for the creation of effective synergetic brews in a wide range of arts, disciplines, and natural phenomena. To the extent that these examples are convincing, they support the generality of the present theory. An additional goal of Part 9 was to show how the descriptive profiles of responses termed “aesthetic” depend on art form and discipline, social context, and culture, in line with the empirical semantic approach described in Appendix B, The paragraph titled Fuzzy Boundaries of the Aesthetics Concept.

### Mainly or Purely Mental Synergetic Brews

The observation that the component elements of synergetic brews always include a blend of exteroceptive and internal (covert) stimuli (Sections 1.8, 2.5, and Part 5) raises the question of whether synergetic brews that have no exteroceptive components at all, that is, all of whose ingredients are covert or mental, can evoke aesthetic responses. Can the elements of a synergetic stimulus consist entirely of covert behavior—of purely mental events? The initial operant component (looking, listening, etc.) would then be absent, but this difference may prove unimportant. If aesthetic responses can be evoked by purely mental synergetically interacting elements, then the creation of the synergetic brews can also be entirely covert and private. Tantalizing, but beyond the scope of the present analysis, is the possibility of extending the concept of purely mental synergetic brews to subaesthetic events.

Instances of aesthetic effects generated by synergetic brews in which covertly generated stimuli predominate are: the chess player who finds a beautiful move upon having (mentally) assembled several concepts regarding the position, and Archimedes who makes the 12-link connection after some pondering, culminating in his “Eureka!” (Section 6.3). Creative writers, composers, and artists similarly often do the heavy lifting mentally, and execute the overt outcome of the process (the painter’s brushstrokes, the composer’s writings, the author’s keystrokes) only after the mental phase has been completed.

### Methodological Issues

The challenge posed by the proposed research strategy is to devise laboratory models in which the effects of relevant variables can be measured and interpreted. The issue would always be what a particular laboratory model can tell us about things outside the laboratory. In the context of aesthetics, this issue takes the form of whether study of the model will increase our understanding of some of the phenomena we refer to as aesthetic—how they are generated, how we can categorize them, measure them objectively, and how we can predict and control them.

The value of verbal reports by members of any semantic community regarding aesthetic responses is always limited by the wide diversity of priming histories and cultural factors among the group’s members. For instance, it is rare that any group’s “standards of beauty” (e.g., paintings displayed in a museum, or a particular piece of music) will evoke similar aesthetic responses from all or most of its members.

### Alternative Research Strategies

One way to address this research challenge is to go to single-subject designs where individual participants are used as their own controls: by asking participants to identify stimuli that evoked genuine aesthetic responses *for themselves* in the past, and then using those stimuli in the experiments. This method ensures that the participant has or had the required priming history for responding to that stimulus aesthetically. Since the stimulus may have lost some of its former aesthetic impact for the participant over time, refreshment techniques may be required to restore and maintain its effectiveness for the experiment.

FMRI technology promises to become a source of measures that correlate, potentially at a detailed level, with different types of aesthetic responses, as for instance, in different art forms, disciplines, or types of synergetic brews. FMRI activity patterns, in conjunction with verbal reports and behavioral data, may lead to categorizations of such patterns according to independently established functions of the neural structures involved.

### Observations and Conclusions

- Responses we tend to describe as aesthetic have an emotional component, and this component is usually surprise tinged.
- The stimuli that evoke aesthetic responses involve synergetic interactions among elements.
- Aesthetic phenomena have their evolutionary roots in events that once had biological significance, like alarms or opportunities.
- Stimuli that evoke aesthetic responses tend to be positively reinforcing, in ways that relate to behavioral and biological factors.
- A limited number of concept-manipulation devices is sufficient for the creation of the synergetic brews that generate aesthetic and related emotional effects in the more than 200 examples drawn from 17 arts, disciplines, and natural phenomena.
- Aesthetic phenomena may be special cases of a far more widespread universe of behavior—our continuous stream of subaesthetic reactions.

### Final Comments

As a pervasive and prominent aspect of human behavior, aesthetic responses and synergetic behavioral effects in general, are beckoning targets of scientific inquiry. I believe that the inevitable long-term consequence of such research will be a transformation of the concept of aesthetics itself. Many concepts and constructs, especially the fuzzy ones, start to disintegrate when you shine a bright light on them. That is how science advances. Just as the fuzzy concept of fire has given way, in scientific discourse, to concepts like ignition, combustion, fuel, and oxidation, so must fuzzy concepts like aesthetics and beauty be expected eventually to give way to ones that are more useful in a scientific analysis. The goal should be to bring aesthetic and synergetic phenomena into mainstream biological and behavioral science where they belong.

On a personal note, my interest in aesthetics awoke during my teenage years as an outgrowth of my immersion in the arts. When my Columbia University professors led me to see that behavioral science held the keys to a fuller understanding of aesthetics, I knew that behavioral science would be my life’s work.

Some of the additional things I learned during the 60-year detour I took prior to my current return to those roots helped me write this article. I am hopeful that the forthcoming commentaries in *The Psychological Record* will help me strengthen the theory to the point where it induces some intrepid pioneers to undertake experimental research forays into the relatively untrodden terrain of the scientific study of aesthetics.

## Appendix A

### Research Topics for Experimental Analysis

The primary purpose of this appendix is to make the point that the present analysis of aesthetics opens the door to systematic experimental analysis. These are some of the more obvious topics that can be addressed:

- The behavioral composition of aesthetic responses
  - Relationship between measures of brain activity and their correlations with observed behavioral events
  - Differentiation of the covert emotional component from the overt manifestation of aesthetic responses
  - Methods and techniques for observing and measuring emotional responses in general, levels of surprise, and aesthetic responses, by means of
    - FMRI, GSR, pupillary dilation, other
    - Rankings, preferences, choices^22^
    - Relative reinforcement value

- The parameters of synergetic interactions
  - Number of elements in the synergetic brew
  - Sensory modalities of the elements
  - Presence or absence of emotionalizing elements
  - Diversity of the sensory modalities (e.g., color and shape vs. color and sound)
  - Effect of enhancing some elements selectively
  - The strength of the concepts, relations, and expectations.
  - Changes in fMRI response patterns as a function of successive exposures to the same stimulus
  - Amount of information conveyed by a synergetic interaction^23^

- Effects of emotionalizing ingredients of the synergetic brew
  - Emotionalization due to history variables versus contemporaneous variables
  - Intensity, recency, and duration of emotionalization
  - Type of emotionalizing variable
- Effects of priming variables
  - Number of prior exposures to elements of the synergetic brew
  - Time since prior exposures to stimulus elements
  - Duration of exposures to stimulus elements
  - Relative amounts of discrimination training that stimulus elements have received
  - Form and type of priming elements have received

- Effects of potentiating and interfering factors
  - Presence of (distracting) stimuli that can interfere with aesthetic responses: tactile, auditory, visual
  - Stressors like threats, time pressure, physical discomfort
  - Sociocultural variables
  - Experimentally induced anxiety

- Effects of refreshment of aesthetic effects
  - Elapsed time since last prior exposure to the aesthetic stimulus
  - Number and duration of prior exposures
  - Strength of aesthetic response in prior exposures
  - Intervening exposures to interfering stimuli
  - History of the aesthetic response being refreshed
  - Sensory modality of the response being refreshed (visual, auditory, olfactory, abstract)

- Degree of attribute conservation in symmetrical transformations
  - In music: harmonic, melodic, rhythmic, timbre
  - Visual transformations: coloristic, textures/patterns, intensity, design details
  - In poetry: rhymes, symmetries of meter or of structure, as in stanzas
  - In verbal concepts: symmetrical transformations of meaning

- Effects of repetition on the aesthetic response
  - Numbers of repetitions of the stimulus
  - Habituation and extinction effects of repetitions
  - Effects due to attention-evoking properties of repetition

- Effects of parsimony
  - Modeling and measurement of parsimony: videogame models of power amplification
  - Level (amount) of power amplification
  - Type of power amplification (speed, force, time, leverage, distance)

- Artificial creation of aesthetic effects by use of the devices
  - Can aesthetic effects be generated artificially?
  - Can they be generated by the creation of surprise via nonconfirmation of laboratory-induced expectations?
  - Can models or algorithms be created for the synthesis of double meanings? incongruities? contradictions?

- Other possible research topics
  - Are there genetically encoded aesthetic responses to certain synergetic brews? If so, what are these and what do they have in common?
  - What factors affect the degree to which the interaction of elements within multisensory or cross-modality brews will be synergetic?
  - What variables make some unrealized expectations (i.e., surprises) reinforcing and others not?

- How does the aesthetic response depend on the instructional or informational value of the nonconfirmation of an expectation?
- Are reinforcing effects of creative acts a function of the number of links in multiple-link chains that comprise the new connection? 24
- Are the aesthetic and reinforcement effects of synergetic brews a function of the number of elements comprising the synergetic brew?

## Appendix B

### Prior Work on Aesthetics

#### History

The examination of aesthetics has a long and venerable history going back to Pythagoras, Plato, Aristotle, Burke, Kant, Darwin, and others. More recent work on the psychology and origins of aesthetics includes that of Miller (2000, 2001); Dutton (2003); Rolls (2005, 2011, 2014); Jacobsen (2006); Schellekens and Goldie (2011); Davies (2012); Chatterjee (2012); Shimamura (2012); Rusch and Voland (2013); Starr (2013); and Tinio and Smith (2014).

Much of the prior work concerned stimulus attributes of aesthetic phenomena—usually in the context of visual art, music, or some other single discipline—with the focus on “beauty” and perceptual processes. Gustav Fechner (1860) pioneered “experimental aesthetics,” the study of relationships between stimulus attributes and behavior, later carried further by Berlyne (1971, 1974).

Artur Koestler (1964) focused on the *creation* of the aesthetic stimulus and some of its properties. He described *The Act of Creation* in the arts and humor as “bisociation” and the “collision of matrices.” His concept of *matrix* is compatible with the behavioral definition of *concept* (Sections 2.1 - 2.4), and he defined bisociation as “synthesis, collision, amalgamation, integration, blending, or juxtaposition of matrices.” Fauconnier and Turner (2008) developed Koestler’s work into a cognitive theory of “conceptual blending*.*”

#### Neural Correlates

Some of the more recent work on aesthetics has focused on CNS correlates (e.g., Chatterjee, 2012; Kandel, 2012; Kawabata & Zeki, 2004; Rolls, 2014; Rolls & Grabenhorst, 2008; Starr, 2013). The amygdala is involved in the processing of emotions (including aesthetic responses), the learning of emotional responses, the storage of emotional memories, and classical conditioning. It has multiple and elaborate neural linkages to other parts of the brain, especially its immediate neighbors—the hippocampus (the site of long-term memory formation and storage), the nucleus accumbens (a site of positive reinforcement and dopamine release), and the thalamus (Kandel, 2006, 2012; Kawabata & Zeki 2004; Rolls & Grabenhorst, 2008). Neurobiologists’ findings jibe with the observation that emotions, including those involving aesthetic responses, may rival in their complexity those of our verbal and cognitive responses.^25^ Earl Miller’s demonstration that conceptualization and categorization of stimuli occurs in the lateral prefrontal cortex (Kandel, 2012, p. 306) is one of many recent examples of brain mapping of behavioral events related to aesthetics, and of the methodological promise of neuropsychology for its scientific study.

#### Conjectures Regarding Evolutionary Origins

Darwin and others pointed out how the mating rituals of various animal species, and plant pollination dynamics, led to the evolution of some species’ attraction to certain songs, flavors, or colors—possible proto-aesthetic phenomena. The mating rituals of many nonhuman species contain displays that may be evolutionary precursors of our arts—suggesting that our arts evolved, at least in part, as an advertising medium for the performer’s fitness and competence as providers in the procreation enterprise, and as carriers of desirable genes (e.g., Davies, 2012; Dutton, 2003; Flannery, 1993; Miller, 2000; 2001). Rolls (2011, 2014) discussed the evolutionary origins of the reinforcing effects of aesthetic phenomena.

#### Relation to Other Research

Synergetic types of stimulus effects differ from the summative, overshadowing, and other types of effects that have been the subject of traditional research on compound stimuli or stimulus control (e.g., Cumming, Berryman, & Cohen, 1965; Thein, Westbrook, & Harris, 2008). The main differentiating feature of synergetic stimuli is their emotion-evoking transformative effect when their interactions are potentiated. This features also raises different types of research questions (see Appendix A).

#### The Fuzzy Boundaries of the Aesthetics Concept

The diversity of adjectives that our semantic community would usually use to describe the effects analyzed in Part 9, as discussed in Section 9.1, illustrates the fuzziness of the aesthetics concept. Although all the analyzed effects share the same device structures, the term *aesthetic* is not always the first one that comes to mind when we describe them.

The disciplines of empirical linguistics (Glynn & Fischer, 2010; Rieger, 1989) and catastrophe theory (Wildgen, 1983) adopted the approach of “empirical semantics” to address fuzzy concepts like aesthetics. This approach identifies and describes the concept’s constituent descriptive elements and then assigns them weights (in the 0 to 1 range), according to the magnitude of their contribution, thus creating a descriptive profile.

Lofti Zadeh—founder of fuzzy math (Zadeh, 1976, 1978, 1997)—proposed a similar method for the analysis of fuzzy concepts (Zadeh, 1965). He used the expression “grade of membership (characteristic)” for the extent (in the 0 to 1 range) to which each attribute of a fuzzy concept is present, and “membership function” for the resulting descriptive profile.^26^ In the analysis of aesthetic responses, the fuzziness would depend on the semantics of the verbal community.

## Footnotes

1 Every use of the term “aesthetic” should be read as having quotation marks around it, to indicate that the concept is a fuzzy one, defined only by how it is used by a particular semantic community, as explained further in Section 9.1.

2 The terms *emotion* or *emotional* will be used throughout only in the sense of a reaction to a stimulus, rather than a state. There is no need here to identify a particular category of emotional reaction (e.g., LeDoux, 2012), or to seek a precise definition.

3 Operant behavior (sometime called instrumental) operates on the environment and is susceptible to being shaped by its consequences.

4 As used here, the term *audience* refers to one or more viewers, listeners, readers, or observers.

5 The term *Works* includes graphic visual arts, sculpture, architecture, music, poetry, literature, performance arts, film, videogames, humor, culinary art, and so forth.

6 The terms *schema, matrix, category,* and *frame* (as in relational frame theory; Hayes, Barnes-Holmes, & Roche, 2001) are variously used in ways similar to *concepts* and their relations.

7 Any boundaries on the range of behavior to which the discrimination-generalization definition of “concept” applies would be arbitrary. I thus feel free to transcend the usual verbal-cognitive usage.

8 The reinforcing power of daredevil activities, videogames, and gambling may be due in part to the biological utility of learning to manage danger through experience gained when expectations generated in such situations are or are not realized, regardless of whether the dangers are real, simulated, or vicarious. The appeal of movie thrillers may have similar origins.

9 Perhaps an operant version of the classical Rescorla and Wagner (1972) model, where “expectation” refers to the behavioral effects of a learning history with respect to certain stimuli.

10 The entertainment industry makes extensive use of the reinforcing properties of power amplification by offering vicarious identification with superheroes, play with superweapons, flying through the air, and supervehicles that create the illusion of power amplification.

11 Although any stimulus can come to elicit conditioned emotional responses, the emotion-tinged linkages that can form with odors and musical stimuli are particularly strong and durable (Levitin, 2006).

12 Certain biologically significant concepts like nourishment-related stimuli, various danger signals, faces, and voices may be learned so rapidly as to seem innate. The speed and ease with which learning occurs defines the continuum from early learning (at one extreme) to full genetic encoding at the other.

13 Responses based on universally shared priming may be responsible for the common illusion that such qualities as beauty are attributes of the object, like size or shape.

14 It goes without saying that the individual’s method of listening, viewing, or study determines the effect of such exposure.

15 Equivalence researchers have called it “nodal distance” (Fields, 2015; Fields & Verhave, 1987).

16 A closely related finding is that the greater the number of relations within English text (meaningfulness), the faster and more easily it can be understood and copied (Cook, 1972; Shannon, 1951).

17 The many ways in which nonrealization of expectations creates surprise and emotional impact in music are analyzed in more detail and at greater length in Levitin (2006, Chapter 8).

18 Though it was Wagner who gave the *Leitmotif* device its name and used it in his operas, the device itself had already been used by Mozart (e.g., the *Commendatore* theme in *Don Giovanni*), and by many others since.

19 This film was a collaboration between the Walt Disney studio of the 1930s and 1940s, the musicians Leopold Stokowski and Deems Taylor, and eight great classical composers.

20
*Go* is a deep 3,000-year-old strategy board game widely played throughout Asia. Two players take turns placing black-and-white stones on a 19 × 19 grid to surround intersections and capture groups of enemy stones by surrounding them.

21 The role of parsimony was discussed in Sections 4.7 - 4.9, 9.6, and 9.7.
